# Multifaceted framework for defining conservation units: An example from Atlantic salmon (*Salmo salar*) in Canada

**DOI:** 10.1111/eva.13587

**Published:** 2023-09-15

**Authors:** Sarah J. Lehnert, Ian R. Bradbury, Brendan F. Wringe, Mallory Van Wyngaarden, Paul Bentzen

**Affiliations:** ^1^ Northwest Atlantic Fisheries Centre Fisheries and Oceans Canada St. John's Newfoundland and Labrador Canada; ^2^ Bedford Institute of Oceanography Fisheries and Oceans Canada Dartmouth Nova Scotia Canada; ^3^ Biology Department Dalhousie University Halifax Nova Scotia Canada

**Keywords:** conservation units, COSEWIC, endangered species, evolutionary significance, genetic discreteness, genomics

## Abstract

Conservation units represent important components of intraspecific diversity that can aid in prioritizing and protecting at‐risk populations, while also safeguarding unique diversity that can contribute to species resilience. In Canada, identification and assessments of conservation units is done by the Committee on the Status of Endangered Wildlife in Canada (COSEWIC). COSEWIC can recognize conservation units below the species level (termed “designatable units”; DUs) if the unit has attributes that make it both discrete and evolutionarily significant. There are various ways in which a DU can meet criteria of discreteness and significance, and increasing access to “big data” is providing unprecedented information that can directly inform both criteria. Specifically, the incorporation of genomic data for an increasing number of non‐model species is informing more COSEWIC assessments; thus, a repeatable, robust framework is needed for integrating these data into DU characterization. Here, we develop a framework that uses a multifaceted, weight of evidence approach to incorporate multiple data types, including genetic and genomic data, to inform COSEWIC DUs. We apply this framework to delineate DUs of Atlantic salmon (*Salmo salar*, L.), an economically, culturally, and ecologically significant species, that is also characterized by complex hierarchical population structure. Specifically, we focus on an in‐depth example of how our approach was applied to a previously data limited region of northern Canada that was defined by a single large DU. Application of our framework with newly available genetic and genomic data led to subdividing this DU into three new DUs. Although our approach was developed to meet criteria of COSEWIC, it is widely applicable given similarities in the definitions of a conservation unit.

## 1 INTRODUCTION

Our planet is losing biodiversity at an accelerating rate, and this ongoing mass extinction will have wide‐ranging, possibly irreversible, consequences (Ceballos et al., 2020; Desforges et al., 2022). While global action is needed to protect remaining biodiversity, part of this action requires not only identifying vulnerable species, but also identifying units below the species level that contribute to its diversity (Des Roches et al., 2021; Fraser & Bernatchez, 2001). Identifying such units for conservation will not only help prioritize and protect populations that are most at risk, but will also protect the unique intraspecific diversity that can promote species resilience (Schindler et al., 2010) and support ecological and cultural functions (Des Roches et al., 2018; Des Roches et al., 2021). Maintaining diversity also conserves the genetic variation needed for species to respond to changing environmental conditions, such as climate change (Layton et al., 2021).

Defining the appropriate units for conservation within a species has long been a fundamental question in conservation biology (Ryder, 1986), and has significant implications for the status, protection, management, and recovery of species. Over recent decades, different criteria have been proposed for defining conservation units (reviewed by Fraser & Bernatchez, 2001), with many concepts focusing on differentiating units along two axes that encompass isolation and adaptation (Waples, 1991; Waples et al., 2022). In Canada, the federal protection of species falls under the Species at Risk Act (SARA), which establishes and directs a committee of independent experts (Committee on the Status of Endangered Wildlife in Canada; COSEWIC) to identify and assess units for conservation. The criteria used by COSEWIC to delineate conservation units aligns closely with the definition of an evolutionarily significant unit (ESU) originally proposed by Waples (1991). Under COSEWIC, these units are termed “designatable units” (or DUs) and are defined as “representing a unit of Canadian biodiversity that is both discrete and evolutionarily significant,” and guidelines are provided for how these conditions can be met (see COSEWIC, 2020). Briefly, the two main criteria for “discreteness” include: (D1) evidence of heritable traits or markers that clearly distinguish the putative DU from others indicating limited gene flow; and (D2) natural geographic disjunction between putative DUs that severely limits transmission of information between them for an extended time. If a putative DU is found to be discrete based on one or more of the above criteria, the next step is to evaluate support for evolutionary significance. Two main criteria have been identified to infer “evolutionary significance” and include: (S1) strong differences in characteristics that reveal an independent evolutionary trajectory for an evolutionarily significant period, usually associated with separate Pleistocene refugia; and (S2) strong differences from other DUs in adaptive, heritable traits that cannot be practically reconstituted if lost (COSEWIC, 2020).

These guidelines still involve subjective interpretation, as there are various ways that criteria can be met using different approaches, analyses, and data types. In recent years, access to “big data” has raised questions about how to best incorporate new knowledge and forms of data into the existing framework for defining conservation units within COSEWIC (e.g., recent formation of DU Working Group) and beyond (Forester et al., 2022; Forester & Lama, 2022; Funk et al., 2012; Waples et al., 2022). Specifically, advances in genomic analysis technology are providing unprecedented amounts of data for non‐model species that can be leveraged to address criteria of both discreteness (isolation) and evolutionary significance (adaptation) for delineating conservation units in general (Forester et al., 2022; Forester & Lama, 2022; Funk et al., 2012; Waples & Lindley, 2018). While large‐scale genomic datasets have rarely been used for defining COSEWIC DUs, insights from genomic data are being used to guide decisions in other jurisdictions, such as at the federal and state level in the United States (Waples et al., 2022). Given the increasing information that genomic data can provide, it is likely that more COSEWIC wildlife assessments will begin incorporating genomic information as these datasets become more widely available and accessible. This offers new opportunities to use these data to address COSEWIC's criteria for delineating DUs, but a rigorous and repeatable framework using multiple data types is needed to guide the process of DU identification. Such a repeatable framework, especially one that includes a weight of evidence approach, will reduce the risk of over splitting (Coates et al., 2018).

Atlantic salmon (*Salmo salar*; Linnaeus, 1758) is a culturally, ecologically, and economically important species that was previously split into 15 DUs during COSEWIC's assessment of anadromous salmon in 2010, as well as one non‐anadromous extinct DU (COSEWIC, 2010b). These COSEWIC DUs represent only the Canadian populations of Atlantic salmon and are the focus of this study; however, populations in the United States comprise the Gulf of Maine distinct population segment (DPS) which are protected under the Endangered Species Act (US Fish and Wildlife Service and National Oceanic and Atmospheric Administration, 2019). Every 10 years, COSEWIC is required to reassess species, and in the intervening period since the last Atlantic salmon assessment, several genetic and genomic datasets have been amassed (Bradbury et al., 2014, 2018, 2021; Jeffery et al., 2018; Moore et al., 2014) providing both impetus and opportunity to reevaluate the DU structure for the species. Studies using these datasets have largely supported the discreteness of many of the 15 DUs for anadromous Atlantic salmon (e.g., Bradbury et al., 2021; Jeffery et al., 2018; Moore et al., 2014); however, some clear discrepancies exist that require reevaluation.

We developed a framework for identifying COSEWIC DUs for anadromous Atlantic salmon in Canada using a weight of evidence approach that incorporates both genetic and genomic data, as well as additional data types (i.e., life history and climate data) (Lehnert et al., 2023). Atlantic salmon represent an excellent species for this exercise because, although there is a wealth of genetic and genomic resources (Lien et al., 2016), this species is also characterized by complex population structure and life‐history variation across the range (King et al., 2001; Klemetsen et al., 2003; Moore et al., 2014). Prolonged periods of isolation combined with a predisposition for Atlantic salmon to return to natal rivers with a high degree of fidelity (Stabell, 1984) has resulted in hierarchical population structuring at multiple spatial scales, including at the level of continents (Lehnert et al., 2020), regions (Moore et al., 2014), rivers (Bradbury et al., 2018), and even within‐rivers (Miettinen et al., 2021). Given the complexity, the full analyses for this process were extensive and comprehensively detailed as part of the pre‐COSEWIC review of Atlantic salmon conducted by Fisheries and Oceans Canada through the Canadian Science Advisory Secretariat (Lehnert et al., 2023). However, here, we focus on broadening this framework to help inform the process of characterizing conservation units for wildlife species in Canada and beyond. We also provide an in‐depth example on how these methods were applied and impacted DU structure in Atlantic salmon in a previously data limited region of northern Canada. Finally, we discuss important considerations for using genomic data to inform conservation units. While our approach was designed to meet criteria of COSEWIC, given the convergence in the definition of conservation units (Fraser & Bernatchez, 2001; Waples, 1991), our approach can be applied more broadly for genetic‐ and genomic‐informed conservation planning.

## 2 METHODS

### 2.1 Framework overview

**FIGURE 1:**
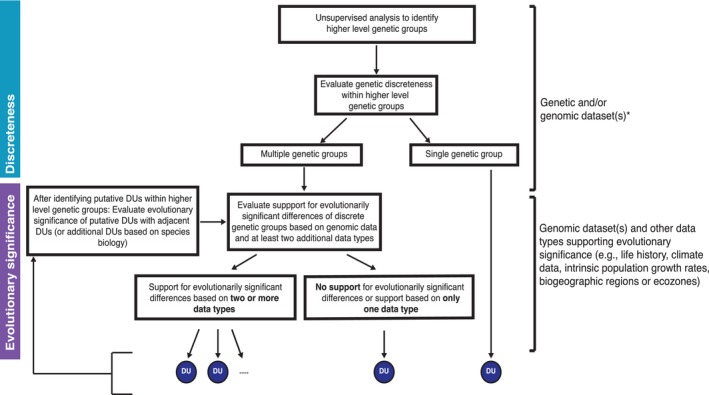
Decision tree framework used to guide the delineation of COSEWIC DUs based on criteria of discreteness and evolutionary significance. The framework incorporates genetic, genomic, and complementary datasets, and is adapted from the decision tree (Figure S1) used for defining DUs of anadromous Atlantic salmon (Lehnert et al., 2023). *Genetic/genomic data are recommended as a focal data source for “discreteness” when available, although we recognize other evidence based on inherited traits (e.g., life history or behavior) or physical barriers that prevent gene flow can also support criteria.

**FIGURE 2:**
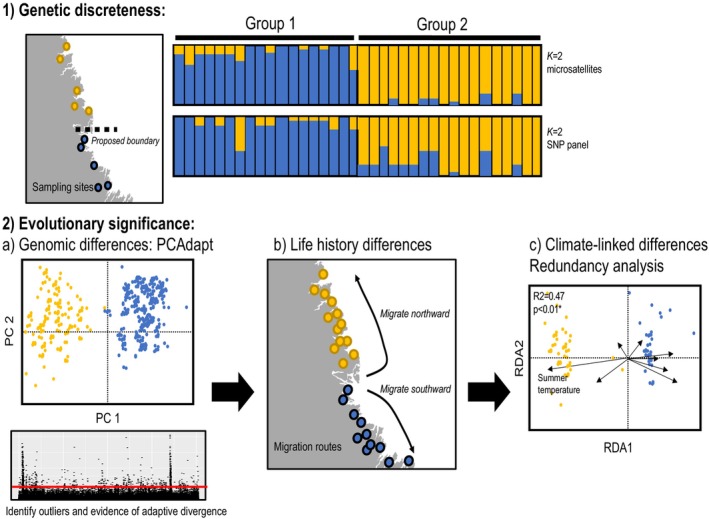
Simplified schematic to illustrate methods used to evaluate genetic discreteness and evolutionary significance for COSEWIC DUs.

We developed a decision tree to provide a repeatable framework to define COSEWIC DUs (see Figure 1), which was adapted from the decision tree used for defining anadromous Atlantic salmon DUs (Figure S1) (Lehnert et al., 2023). Under this framework, the existence of discrete units is first tested, and where present their evolutionary significance is then investigated. Unsupervised analyses of genetic or genomic data are recommended as a starting point for discreteness, such as principal component‐based analyses (Jombart, 2008; Luu et al., 2017) or programs that assign individual ancestry coefficients (e.g., STRUCTURE, LEA, ADMIXTURE) (Alexander & Lange, 2011; Frichot & François, 2015; Pritchard et al., 2000), to first identify higher level structure. Using this hierarchical approach, genetic structure at a finer spatial scale is then examined within higher level groups using genetic and/or genomic datasets that have high geographic coverage. In our example here, we use an individual based clustering approach using the program STRUCTURE for this purpose (see below; Pritchard et al., 2000). After identifying discrete genetic groups, the next step under COSEWIC, as well as for many other jurisdictions, is to evaluate evidence of evolutionarily significant differences between the identified groups. Our framework employs a novel weight of evidence approach for assessing evolutionary significance by first evaluating evidence of genomic‐based adaptation between discrete units, and then comparing other data types that examine evolutionary significance; in the example below, we use life history and climatic datasets. To ensure stable and robust DUs, we required support from at least two of these three datasets to fully support DU criteria. After identifying one or multiple DU(s) within a higher level group, support for evolutionary significance of those DUs from neighboring DUs (in other high‐level groups) must be examined. In the Atlantic salmon example described here, we focus on adjacent DUs where there is greater potential for gene flow and adaptive similarities; however, comparisons may vary depending on species biology. A simple schematic overview depicting the workflow that can support discrete and evolutionarily significant units is provided in Figure 2, and full details of the methods are described here.

### 2.2 Discreteness

#### 2.2.1 Identifying higher level genetic groups

The first step for identifying COSEWIC DUs includes detecting evidence of discreteness. In our framework, a hierarchical approach was used to first identify higher level genetic groups (Figure 1). Given the wealth of genetic and genomic studies in Atlantic salmon, Lehnert et al. (2023) used the previously defined COSEWIC DUs as a starting point to evaluate genetic discreteness within higher level groups (for more details, see Lehnert et al., 2023; Figure S1). While we use Atlantic salmon as an example here, we also broaden this approach to demonstrate that genetic or genomic data can also be used as a starting point for discreteness using unsupervised analyses to evaluate hierarchical structure without a priori knowledge of population structure. Here, we first used a principal component analysis (PCA)‐based approach to examine broad‐scale genetic structure and identify higher level genetic groups. Genomic data from a 220,000 SNP array (see Barson et al., 2015) developed using a targeted, biallelic SNP Affymetrix Axiom array by the Centre for Integrative Genetics (CIGENE, Ås, Norway) were compiled for Canadian populations of Atlantic salmon, and included both data from previously published sources (Lehnert, Bentzen, et al., 2019; Lehnert, Kess, et al., 2019; Lehnert et al., 2020; Sylvester et al., 2018; Watson et al., 2022) as well as unpublished data. Genotyping was performed using the Affymetrix axiom array protocol (Thermo Fisher Scientific), with raw data processed using the Axiom Analysis Suite (AAS) software based on manufacturer's best practices protocol. AAS assigns all SNP assays into different categories based on clustering patterns, and only SNPs that were classified as high quality were used for subsequent analyses. SNPs were filtered using PLINK v1.9 (Purcell et al., 2007) for minor allele frequency (MAF) of 0.05, resulting in retention of 95,043 loci with a genotyping rate of 0.998. Our dataset comprised 105 locations and 4074 individuals (see Table S1). We ran the PCA using the R package *pcadap*t (Luu et al., 2017) to investigate broad‐scale genetic structure with *K* = 6, as little additional variation was explained beyond *K* = 6 (Figure S2). Based on the initial PCA, we identified outliers, removed these loci from the dataset, and reran the PCA to assess population structure based on this “neutral” dataset. To identify outliers, the *p*‐values associated with SNPs were corrected for false discovery rate using the R package *qvalue* (Storey et al., 2015; Storey & Tibshirani, 2003); loci with corrected *q*‐values below 0.05 were considered outliers. The PCA based on the neutral dataset identified genetic groups along the first two PCs (see Results), and to illustrate how our framework was applied, we provide an in‐depth example of our approach within one of these higher level groups. We focus our example on Labrador, as the original Labrador DU (DU‐02) defined by COSEWIC in 2010 encompassed an extensive area covering over 1300 km of coastline from the northern tip of Labrador, and south along the coast to the Napitipi River in Quebec. Given the large size of this geographic region, it was previously suggested that there was substantial potential for smaller DUs within this region, although data were lacking at that time (COSEWIC, 2010b). Therefore, this region provides an excellent case study for our application.

#### 2.2.2 Evaluating genetically discrete groups

After identifying Labrador as a higher level discrete genetic group (see above and Results), our approach next focused on two types of genetic markers to inform discreteness, including microsatellite and SNP datasets. The primary datasets used to evaluate discreteness in Lehnert et al. (2023) were chosen for their broad and high‐resolution geographic coverage across the Canadian range of Atlantic salmon, as each dataset were comprised of thousands of individuals (*n* = 12,064 for 15 microsatellites; *n* = 5703 for 96 SNPs) from almost 200 locations (Lehnert et al., 2023). However, for Labrador specifically, we leveraged a different microsatellite dataset comprised of 101 loci that has higher geographic coverage in this region (see Bradbury et al., 2018) compared to the 15 microsatellite dataset that was primarily used for other regions of Canada (Lehnert et al., 2023). Although microsatellites are expected to behave neutrally and SNPs may not, previous work suggest that both types of data provide largely consistent results when characterizing population structure in Atlantic salmon (Moore et al., 2014). In addition, the 96 SNP panel used here was originally developed based on its ability to discriminate genetic groups of Atlantic salmon based on neutral structure identified by the 15 microsatellite dataset (for details see Jeffery et al., 2018), and thus we expect these data types to provide similar information about neutral genetic divergence.

In Labrador, there are 102 salmon‐bearing rivers within the boundaries of the original 2010 COSEWIC Labrador DU, and our genetic datasets include samples from 34 locations for the 101 microsatellite dataset (*n* = 1433; Table S2) and 45 locations for the 96 SNP dataset (*n* = 1333; Table S3). Similar to analyses described above to identify larger scale genetic groups, the same approaches (e.g., PCA, individual ancestry coefficients) can be applied to evaluate finer scale structure, and may require a hierarchical approach depending on the number of populations and geographic region examined. Additionally, several other population genetic metrics could also be used to evaluate discreteness, such as genetic differentiation (*F*_ST_) or evaluating migration between units. However, we recommend using unsupervised analyses as a starting point, as this does not require prior knowledge of the species' population structure or for groups to be defined a priori. In addition, statistically significant *F*_ST_ values can be generated when gene flow between groups is high. Therefore, these metrics can be useful in conjunction with unsupervised analyses to further corroborate findings.

In our example, the program STRUCTURE was used to evaluate discreteness, which uses a Bayesian clustering approach to identify discrete populations and assigns individuals to populations based on multi‐locus genotype data (Pritchard et al., 2000). Using STRUCTURE v 2.3.4, independent Markov chain Monte Carlo (MCMC) runs were performed separately for each dataset and implemented through the R package *parallelstructure* (Besnier & Glover, 2013). For each run, a burn‐in of 100,000 followed by 500,000 iterations was performed and this was replicated three times for each value of *K* (genetic clusters), which ranged from 1 to 10 for each dataset. To determine support for the number of genetic clusters (*K*), the optimal number of K was determined based on the ∆*K* statistic (Evanno et al., 2005). However, this statistic can be unreliable in complex evolutionary scenarios (Janes et al., 2017), which can often be the case for salmonids. Therefore, using STRUCTURE HARVESTER (Earl & VonHoldt, 2012), we not only considered the ∆*K* statistic but we also examined the plateau in mean LnPr(*X*|*K*) estimates to assess support for the number of genetic clusters (Janes et al., 2017). All STRUCTURE plots were visualized using CLUMPAK (Kopelman et al., 2015) and were visually inspected to confirm the presence of genetic structure.

STRUCTURE results were evaluated to determine the number of discrete genetic groups present. If only a single genetic group was detected (i.e., no genetic structure), the criterion of multiple discrete genetic groups needed to recognize more than one DU would not be met (Figure 1). If multiple genetic clusters were present in the STRUCTURE analyses, the next step in the framework is to evaluate whether any of the discrete genetic groups met criteria of “evolutionary significance” to fully support their recognition as a DU as defined by COSEWIC (Figure 1). Here, it is important to note that we considered criteria for multiple discrete genetic groups met if STRUCTURE analyses identified multiple groups in one or both datasets (microsatellites and/or SNPs). While these datasets can complement each other and generally provide consistent results in the species (Moore et al., 2014), it is also possible they may disagree because of differences in the markers and geographic sampling. If one dataset shows evidence of genetic discreteness and the other one does not, we argue that this does not indicate an absence of structure but may instead suggest that alleles or loci present in one dataset may be important for discriminating populations and warrant further investigation. Finally, although we relied primarily on genetic data to support “discreteness” criteria, we recognize that other evidence of discreteness based on inherited traits, such as life history or behavior, or physical barriers that prevent gene flow also support discreteness criteria and can be included in our framework; however, genetic data are recommended as a focal data source when available.

### 2.3 Evolutionary significance

#### 2.3.1 Genomic data

Following identification of genetically discrete units, the next step was to evaluate support for “evolutionary significance” of these units (Figure 1). Here, we first used genomic data to inform evidence of genomic‐based putatively adaptive differences between the units. High‐density genomic data were compiled for Canadian populations of Atlantic salmon as described above using a 220,000 SNP array (see *Identifying higher level genetic groups*); within the original Labrador DU, data were available for 19 locations from previously published (Sylvester et al., 2018) and unpublished sources (Table S1). Genomic data were analyzed using the R package *pcadapt* (Luu et al., 2017) which uses a PCA‐based approach to detect loci under selection. We used *K* = 2 in *pcadapt* and used a MAF cutoff of 0.05, resulting in retention of 85,745 SNPs for the analysis. In this approach, we determined if the genetically discrete groups identified by STRUCTURE show genomic differences, and determine which loci contribute to these differences. As above, *p*‐values for SNPs were corrected for false discovery rate using the R package *qvalue* (Storey et al., 2015; Storey & Tibshirani, 2003), and loci with *q*‐values below 0.05 were considered outliers. Results were visualized and inspected by plotting *q*‐values on a Manhattan plot generated with the R package *qqman* (Turner, 2014). To meet criteria of “evolutionary significance,” evidence was required that loci contributing to differences are associated with adaptation. We inferred such adaptive associations based on various lines of evidence that relate to the functional role of outlier loci (see Table 1). For example, after identifying outlier loci, we examined functional enrichment of gene regions associated with the differences between genetic groups (relating to one line of evidence outlined in Table 1). Following similar methods as Lehnert et al. (2020), we conducted gene ontology (GO) enrichment analysis based on GO annotations for Atlantic salmon from SalmoBase (Samy et al., 2017). We identified a “reference” (based on all SNPs) and “outlier” (based on outlier SNPs only) set of genes using BEDTOOLS (Quinlan & Hall, 2010), where for each dataset, we extracted genes that were within 10 kb of the SNPs. Next, the R package *topGO* (Alexa & Rahnenfuhrer, 2016) was used to test for overrepresentation of GO biological processes in the outlier dataset relative to the reference. We used a node size of 5 and the “weight01” algorithm to account for structural relationships among GO terms, and an alpha level of 0.05 was chosen to evaluate significance of overrepresented processes. Overrepresented processes were summarized visually using the Web server REVIGO (Supek et al., 2011). In addition to our formal genomic analyses, we also incorporated information from prior genomic studies conducted in Labrador to further support our assessment (Lehnert, Bentzen, et al., 2019; Sylvester et al., 2018) which related to other lines of evidence in support of genomic‐based differences (see Table 1). These additional lines of evidence relate to outlier loci being located near or within gene(s) known to play a role in adaptation including climate adaptation, as well as outlier loci being located within structural variants that have been associated with putative adaptation (see full details in Table 1).

**TABLE 1:** Description of different lines of evidence used to infer genomic‐based adaptation of a designable unit (DU) to support COSEWIC's criteria of “evolutionary significance,” with specific example related to Atlantic salmon (*Salmo salar*).

Evidence of genomic‐based adaptation	Description and examples for Atlantic salmon
Outlier loci are found within/near genes and this set of genes is associated with overrepresented biological processes	Evidence that loci that contribute to differences between genetically discrete groups are located within or near genes with putative functions. This can be accomplished by examining biological processes associated with a set of genes using gene ontology (GO) term enrichment. This approach can help determine what types of biological processes are overrepresented by the set of genes (those associated with outlier loci) relative to the genomic background *Atlantic salmon*: Studies using GO term enrichment analyses have been used to help understand functional differences between salmon populations or groups that may contribute to adaptation (Lehnert et al., 2020; Wellband et al., 2019; Wringe et al., 2018)
Outlier loci are located within/near gene(s) with known role in adaptation and/or that are associated with climate	Evidence that loci that contribute to differences between groups are located within or near genes that play a role in adaptation for the species. This can be accomplished by reviewing the genetic/genomic work for the species to identify genes that have been previously associated with specific traits or environmental adaptation. Alternatively, genome‐wide association studies or genotype–environment associations can be performed to identify these candidate genes *Atlantic salmon*: Several genes are known to play a role in adaptation in Atlantic salmon. These include (but are not limited to) *vgll3* that influences age‐at‐maturity (Barson et al., 2015), *six‐6* which is associated with age‐ and size‐at‐maturity, river catchment size, and run timing (Cauwelier et al., 2018; Pritchard et al., 2018; Sinclair‐Waters et al., 2020), and major histocompatibility (MHC) genes which are associated with immune function and temperature (Dionne et al., 2007). Other genes that are associated with adaptive phenotypes include growth rate (Gutierrez et al., 2015), immune function (Kjærner‐Semb et al., 2016), and carotenoid pigmentation (Helgeland et al., 2019). In addition, genetic markers associated with climate adaptation have also been identified in Atlantic salmon, and generally, these associations are found to be polygenic (Jeffery et al., 2017; Sylvester et al., 2018). Genes associated with known functional traits and adaptation in other salmonids may also provide insight in Atlantic salmon, as recent evidence suggests a role for the same gene influencing the same trait across Pacific and Atlantic salmon species (Waters et al., 2021)
Outlier loci are located within known structural variants that are associated with adaptation	Evidence that loci that contribute to differences between groups are found in structural variants associated with adaptation in the species. With advances in genomics, it is becoming clear that structural variants like chromosomal rearrangements often underlie complex phenotypes (Mérot et al., 2020; Wellenreuther & Bernatchez, 2018). These variants result in changes in chromosome structure, influence the order and position of genes, can suppress recombination, and can influence gene expression. For example, in salmonids, a chromosomal rearrangement influences the migratory ecotypes (Rainbow Trout vs Steelhead) in *Oncorhynchus mykiss* (Pearse et al., 2019) *Atlantic salmon*: Known chromosomal rearrangements associated with adaptation include chromosomal fusions, translocations, and inversions. Differences in a chromosomal translocation between Atlantic salmon chromosomes Ssa01 and Ssa23 are associated with historical European introgression in North American populations (Lehnert et al., 2019a) and evidence suggests that this translocation is under selection and associated with climate adaptation (Watson et al., 2022). Variation in a chromosomal fusion between Ssa08 and Ssa29 has also been identified across North American populations (Lehnert, Bentzen, et al., 2019), and this fusion has been associated with climate variation (Wellband et al., 2019). Additionally, a 3 Mbp inversion has been identified on Ssa18 that is associated in precipitation and drainage area in salmon populations in eastern Canada (Stenløkk et al., 2022)

#### 2.3.2 Life history and climate‐linked differences

Additional criteria for evidence of evolutionary significance included (1) life‐history differences and (2) climate‐linked differences that are likely to give rise to local adaptation. Anadromous Atlantic salmon spend the first part of their life cycle in fresh water and later migrate to feeding grounds in the marine environment, before returning to fresh water to spawn (Klemetsen et al., 2003). There is extensive variation in the timing of these life events (e.g., age at seaward migration [known as smolt age], age at maturity, and years spent at sea) which contributes to the diversity and uniqueness of life‐history characteristics in the species (Klemetsen et al., 2003). While environmental factors can shape differences in these traits, genetic factors also play a role in determining life‐history differences (Barson et al., 2015; Cauwelier et al., 2018; Páez et al., 2011). For the Labrador DU, we compiled data on life‐history characteristics based on previous syntheses (Chaput et al., 2006; DFO & MNRF, 2009; Hutchings & Jones, 1998) and recently compiled pre‐COSEWIC data (Kelly et al., in prep). Generally, life‐history data are limited and sparse in this region of Canada, but information for these rivers includes sea age, run timing, size at maturity, and smolt age.

As noted above, environmental differences can also be important for shaping life‐history variation and local adaptation (King et al., 2001; Klemetsen et al., 2003; Metcalfe & Thorpe, 1990; Schaffer & Elson, 1975). In our study, we evaluated climate‐linked differences by extracting 19 bioclimatic variables (see Table S4) from WorldClim (Fick & Hijmans, 2017) using the R package *rbioclim* (Exposito‐Alonso, 2017) for potential salmon‐bearing rivers in the geographic region. The 19 bioclimatic variables were generated from monthly temperature and rainfall data over several decades. These precipitation and temperature variables relate to annual trends, seasonality, and extreme environmental conditions (Fick & Hijmans, 2017). The geographic coordinates of each river were used for data extraction and these locations were accessed from the North Atlantic Salmon Conservation Organization (NASCO) river database. A redundancy analysis (RDA) was performed using the R package *vegan* (Oksanen et al., 2017), with bioclimatic data (scaled) as the response variables and genetic group (based on genetic discreteness) as the constraining factor. The RDA was used to identify whether significant climate‐linked differences exist between the discrete genetic groups and which climatic variables contribute to such differences. Significance of the model was tested using an ANOVA‐like permutation test, and a significant model (*p* < 0.05) was used to infer evidence of local adaptation driving differences between the genetic groups, thus supporting criteria of “evolutionary significance.”

Following our decision tree framework, we required support for at least two of the three data types (genomic, life history, and climatic) to provide a weight of evidence to fully support evolutionary significance of DUs (Figure 1). Information on ecological differences were also evaluated (DFO & MNRF, 2009), such as any data relevant to the species environment and ecology that likely drive local adaptation (e.g., river gradient and fish community), and incorporated here to further support inferences of significance. These data were not explicitly required based on our decision tree but can be used to add further support to the DUs.

Finally, the last step after identifying one or multiple putative DU(s) within the higher level group was to evaluate evolutionarily significant differences between these putative DUs and any neighboring DUs in other higher level genetic groups. At this stage in the framework process, discreteness between the putative DUs was already established based on initial unsupervised analyses, and thus only criteria of evolutionary significance needed to be addressed following the same approach outlined above. In the case of Atlantic salmon, significance of DUs was compared against adjacent DUs, consistent with the biology of the species. Specifically, for Labrador, adjacent DUs to the south (Quebec Eastern North Shore DU) and north (Nunavik DU) were evaluated; however, their significance has been previously supported in prior evaluation of COSEWIC DUs for Atlantic salmon (COSEWIC, 2010b), and we do not go into full details here (see Results). Instead, our focus is to determine the discreteness and significance of any newly identified DUs.

## 3 RESULTS

### 3.1 Genetic discreteness

#### 3.1.1 Identifying higher level genetic groups

PCA of neutral genomic data (i.e., with outliers removed) across Canadian Atlantic salmon populations revealed clear separation of Labrador populations from other regions in Canada. These populations corresponded to the previously recognized Labrador DU (COSEWIC, 2010b; Figure S3), and we applied our framework to this higher level discrete genetic group.

#### 3.1.2 Evaluating genetically discrete groups

**FIGURE 3:**
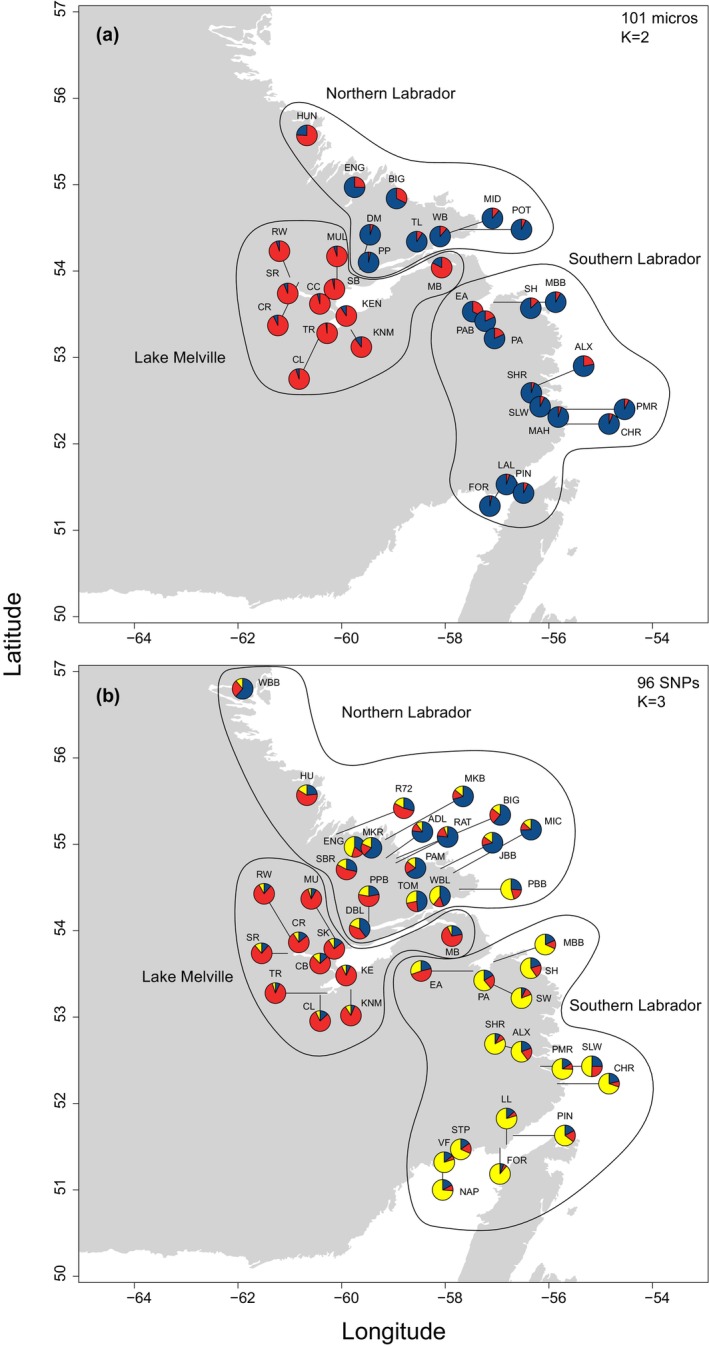
Results of STRUCTURE analyses for the (a) 101 microsatellite dataset and (b) 96 SNP dataset for Atlantic salmon (*Salmo salar*) in Labrador. Pie charts show population membership to genetic clusters. Additional structure was observed beyond *K* = 3 and shown in Figures S5–S7. Reproduced with permission from Lehnert et al. (2023).

Within the Labrador group, following our hierarchical methodology, our genetic analyses determined that three major genetically discrete groups were present (see below; Figure 3), which has also been demonstrated in other studies (Bradbury et al., 2018, 2021). The three groups included (1) Northern Labrador, (2) Lake Melville, and (3) Southern Labrador. These groups are divided in central Labrador near Lake Melville, which is a large, deep (over 200 m) embayment (3069 km^2^) with an extended estuary. Rivers north and south of this embayment represent the Northern Labrador and Southern Labrador genetic groups, respectively, with the rivers located within the embayment representing the Lake Melville genetic group (see Figures 3 and S4).

**FIGURE 4:**
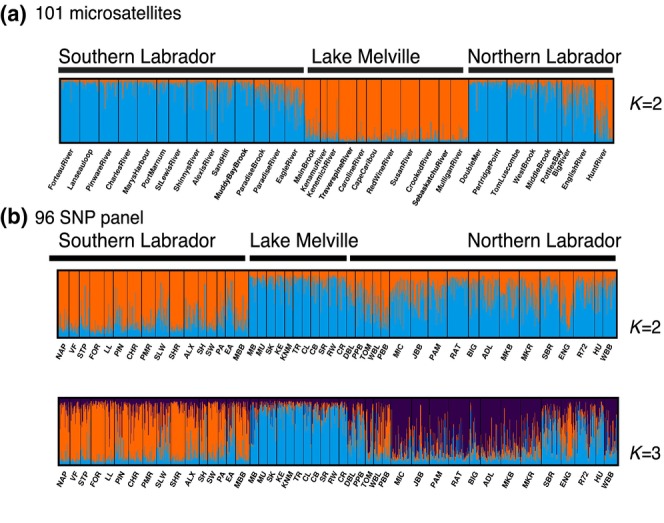
Results of STRUCTURE analyses for the (a) 101 microsatellite dataset and (b) 96 SNP dataset for Atlantic salmon (*Salmo salar*) in Labrador. STRUCTURE plots show individual membership to genetic clusters for different values of *K*. Additional structure was observed beyond *K* = 3 and shown in Figures S5–S7. Populations are ordered from south to north.

Using the 101 microsatellite dataset, the optimal number of genetic clusters (*K*) was 2 based on the ∆*K* statistic (Evanno et al., 2005) (see Table S5), which clearly separated sites in Lake Melville from other sites in Labrador (Figures 3, 4, and S4). Analysis of the 96 SNP dataset revealed that the optimal number of genetic clusters was also 2 (see Table S5), but this separated sites in Southern Labrador from the rest of Labrador (Figures 3, 4, and S5). Additional structure was supported, where at *K* = 3, sites were separated into three clusters primarily corresponding to Southern Labrador, Lake Melville, and Northern Labrador (Figures 3 and 4).

While some evidence of admixture appears to be present at *K* = 2 and *K* = 3, these patterns more likely reflect an artifact of the STRUCTURE program attempting to assign membership to a limited number of clusters which is commonly seen in other salmonid studies due to the hierarchical nature of their genetic structure (Vaha et al., 2007). Indeed, for both datasets, additional clustering of individual rivers and geographic regions was apparent at higher values of K (Figures S5–S7), and this was supported by the plateau in mean LnPr(*X*|*K*) (Figure S7). For example, for the microsatellite dataset at *K* = 10, approximately five clusters were present in Southern Labrador, three clusters in Northern Labrador, and two clusters in Lake Melville (Figure S6). Based on these analyses and our decision tree, criteria for multiple genetic clusters (discreteness) within Labrador are met. While the genetic datasets highlight different axes of population differentiation, there is clear evidence of the discreteness of Lake Melville from the rest of Labrador, as well as discreteness between populations north and south of Lake Melville (Figures 3 and 4). Genetic structure in Atlantic salmon is hierarchical and complex, and this is highlighted by the results here, where structure occurs at multiple spatial scales. In our framework, we focus on the larger geographic breaks to evaluate significance criteria rather than at the river or local scale as evidence of evolutionary significance is often lacking at finer spatial scales (see Lehnert et al., 2023), and in this case, datasets support genetic differences between three major geographic regions in Labrador.

### 3.2 Evolutionary significance

#### 3.2.1 Genomic data

**FIGURE 5:**
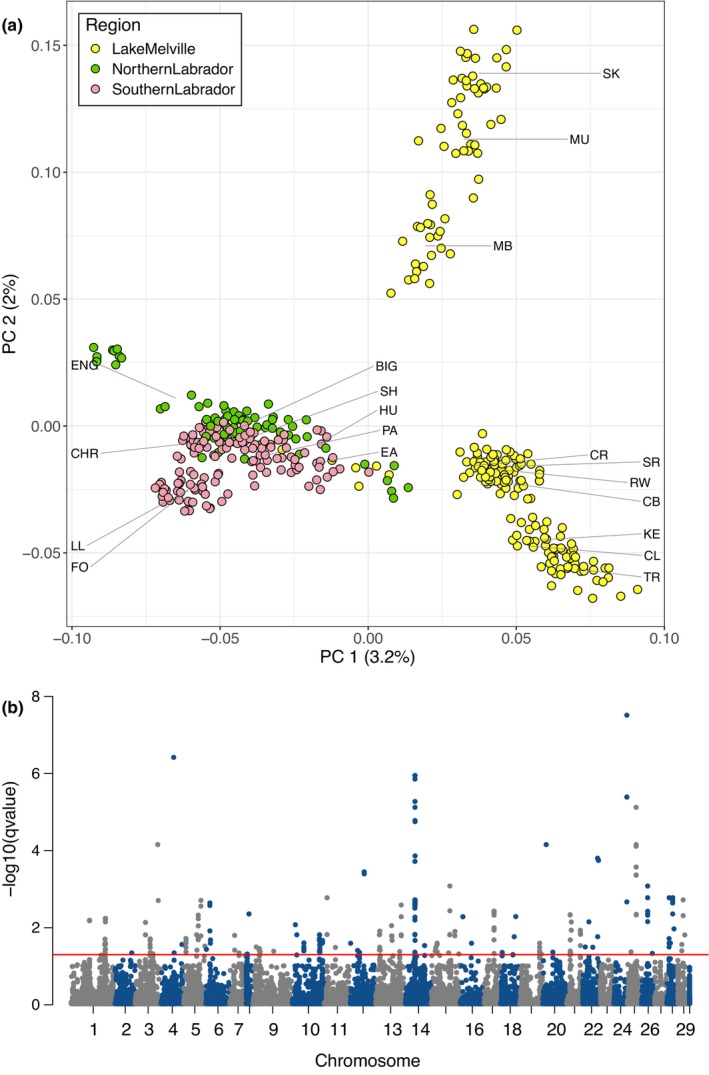
(a) PCA separates populations in Lake Melville from those along the coast of Labrador on the first PC axis using genomic data (85,745 SNPs; MAF > 0.05; *K* = 2). The second PC axis further separates populations within Lake Melville. The mean PC 1 and PC2 values for each population are indicated by lines with their site code provided (see Table S1). (b) Manhattan plot of genomic data where red line indicates genome‐wide significance (*q* < 0.05).

Using *pcadapt*, populations within the Lake Melville genetic cluster were clearly separated from coastal Labrador sites (Southern and Northern Labrador) along the first PC axis (Figure 5). Additional separation along the second PC axis further divided three locations within the Lake Melville system (Main Brook, Mulligan, Sebaskachu) from other sites in Labrador. A total of 314 loci significantly contributed to the differentiation on both PC axes (*q* < 0.05) and these loci were distributed across 27 of the 29 Atlantic salmon chromosomes (Figure 5b) based on the original genome assembly for the species (ICSASG_v2). Gene ontology analyses demonstrated that 86 biological processes were significantly (*p* < 0.05) overrepresented based on the outlier dataset, with a large proportion of processes related to “fatty acid homeostasis” (see Figure S8). Additional evidence supporting genomic‐based putatively adaptive differences among Labrador populations were summarized from recent publications (Lehnert, Bentzen, et al., 2019; Sylvester et al., 2018). This includes evidence of genetic–environment associations delineating coastal Labrador from Lake Melville populations, where precipitation, temperature, and habitat variables were important for differentiating these regions (Sylvester et al., 2018). In addition, a higher frequency (2X) of a European‐type chromosomal rearrangements (Ssa01 and Ssa23 non‐translocation) was found in Lake Melville compared to coastal Labrador (Lehnert, Bentzen, et al., 2019) (see Table S6). Variation in the rearrangement is associated with secondary contact and historical introgression from European Atlantic salmon that occurred near the end of the last glacial maximum as salmon were recolonizing their contemporary range (Lehnert, Bentzen, et al., 2019; Rougemont & Bernatchez, 2018). Further, recent studies suggest this rearrangement is under selection and linked to climate adaptation (Lehnert, Bentzen, et al., 2019; Watson et al., 2022). Therefore, within the Labrador group, genomic data analyses indicate that loci contributing to population differences are associated with putative adaptation based on all three lines of evidence in Table 1.

#### 3.2.2 Life history and climate‐linked differences

Genomic data clearly support evolutionary significance of the Lake Melville system from coastal Labrador, but as part of the weight of evidence process, we also used life history and climate‐linked differences to support further splitting of Labrador based on all three discrete genetic groups. Life‐history differences include differences among all three groups in age‐at‐maturity and run timing, as well as differences between Northern Labrador and one of the other groups in size‐at‐maturity and smolt age (see summary in Table 2).

**TABLE 2:** Information used to support life history and ecological divergence for Atlantic salmon (*Salmo salar*) populations in Labrador, Canada based on previous syntheses and a recent pre‐COSEWIC review.

Life‐history trait divergence	
*Age‐at‐maturity*: Increase in age‐at‐maturity with increasing latitude	Recent data from the Labrador Food, Social, and Ceremonial (FSC) fishery (2017–2019), suggest younger age‐at‐maturity in Southern Labrador, followed by Lake Melville, with the older salmon in Northern Labrador based on sea age for first time spawners (Kelly et al., in prep) Data suggest older age‐at‐maturity in Northern Labrador compared to other regions based on the incidence of one‐sea‐winter (1‐SW) salmon (DFO & MNRF, 2009) Reported older age‐at‐maturity (sea age) for a Northern Labrador population (1.75 years) compared to Southern Labrador populations (range: 1.03–1.16 years) (Hutchings & Jones, 1998)
*Run timing*: Differences in run timing between three genetic groups	Earliest run timing in Lake Melville, followed by Southern Labrador, and with later run timing reported in Northern Labrador (DFO & MNRF, 2009)
*Size‐at‐maturity*: Larger size‐at‐maturity in the north	Size (length) of 1‐SW and 2‐SW salmon was larger for a Northern Labrador population (57.8 and 76.6 cm, respectively) compared to Southern Labrador populations (53.2–54.4 cm and 72.9–74.7 cm) (Hutchings & Jones, 1998)
*Smolt age*: Younger smolt age in Lake Melville	Some evidence to support that Lake Melville has younger smolts compared to coastal Labrador, but data are limited (Kelly et al., in prep; Chaput et al., 2006)

Ecological differences	
*Salmonid community structure*: Differences in salmonid community between the three genetic groups	Northern Labrador rivers are dominated by Arctic charr (*Salvelinus alpinus*). Lake Melville is mainly dominated by Atlantic salmon and sea‐run brook trout (*S. fontinalis*). All three of these salmonid species are represented equally in Southern Labrador populations (DFO & MNRF, 2009)
*River gradient*: Differences in river gradient between the three genetic groups	Lake Melville has the lowest gradient, followed by Southern Labrador, with the highest gradient found in Northern Labrador (DFO & MNRF, 2009)

**FIGURE 6:**
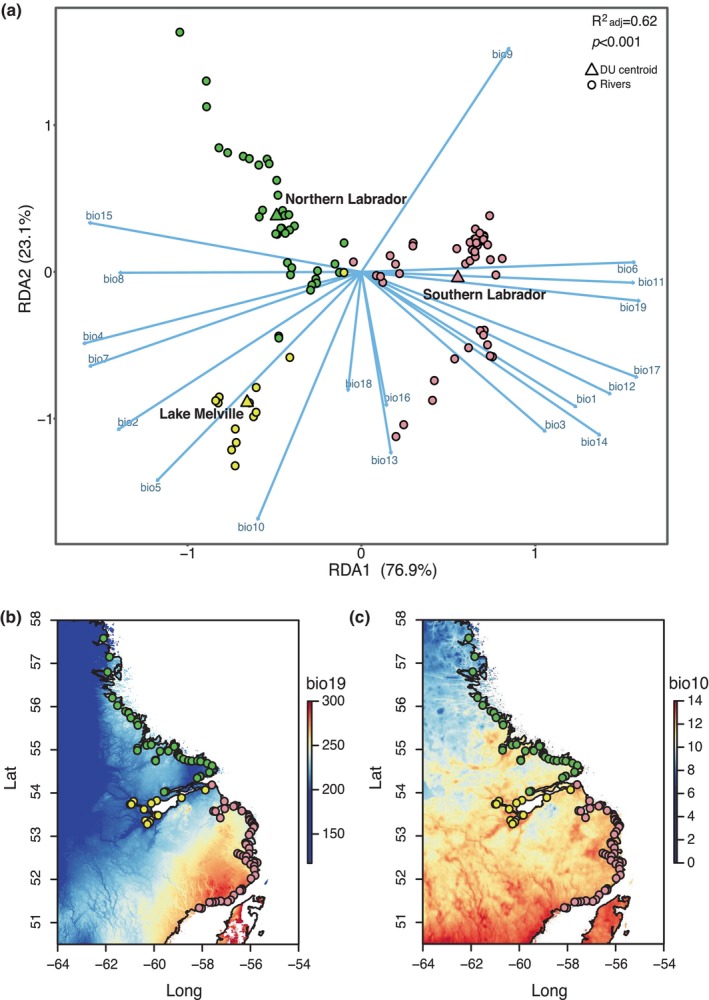
(a) Redundancy analysis (RDA) using bioclimatic data for all salmon‐bearing rivers in the Labrador DU as the response and putative DU groups (three genetic clusters) as the constraining variable. The three putative new DUs include: Northern Labrador (green), rivers draining into Lake Melville (yellow), and Southern Labrador (pink), and centroids of DU groups are indicated by a triangle, with points representing each river. Arrows indicate loadings of bioclimatic variables. Maps of bioclimatic variables (b) bio19 (precipitation of coldest quarter; units mm) and (c) bio10 (mean temperature of warmest quarter; units °C), representing the top loading variables on RDA axis 1 and 2, respectively. Note that in panel (b), values >300 were excluded (shown as white) for visualization, but these only occurred outside of the Labrador region on the map.

Further, climatic data for Labrador also supports differences between the three genetic clusters based on redundancy analysis (RDA) (Figure 6). ANOVA on the RDA showed the model to be significant (*p* < 0.001) with an adjusted *R* ^2^ of 0.62. Both RDA axes were significant (*p* < 0.001), with RDA axis 1 separating Southern Labrador from other regions, and explaining 76.9% of the variance in the model. The highest loading variable on RDA axis 1 was precipitation of the coldest quarter (bio19) (see Figure 6b). RDA axis 2 separated Lake Melville from Northern Labrador, explaining 23.1% of the variance, with mean temperature of the warmest quarter (bio10) loading highest on this axis (see Figure 6c). Full details on RDA results are provided in the Supplement. These results support clear differences in climate that are linked to the three genetic groups which can drive local adaptation.

Finally, differences between the three regions were also supported by ecological differences (DFO & MNRF, 2009), including differences in salmonid community structure across the three regions, as well as differences in river gradient (see Table 2). For example, rivers in the Northern Labrador DU are dominated by Arctic charr (*Salvelinus alpinus*), whereas the Lake Melville DU is mainly dominated by Atlantic salmon and sea‐run brook trout (*S. fontinalis*), and all three of these salmonid species are represented equally in Southern Labrador DU (DFO & MNRF, 2009). In addition, the Lake Melville DU has the lowest river gradients, followed by Southern Labrador DU, with the highest river gradients found in the Northern Labrador DU (DFO & MNRF, 2009). While these factors are not directly included in our decision tree, these variables, like river gradient and interspecific interactions, are relevant to influencing adaptive variation in salmon populations (He et al., 2018; Pritchard et al., 2018; Wellband et al., 2019), and are thus reported here as additional support for the DUs.

Overall, our analyses suggest that there are three discrete and evolutionarily significant units (i.e., DUs) within Labrador, which include (1) Northern Labrador, (2) Lake Melville, and (3) Southern Labrador, and full details supporting this determination are summarized in Table S7. Discreteness of these three DUs is supported by genetic data, where different datasets highlighted different axes of differentiation, but both datasets supported the three (and more) groups. Evolutionary significance based on genomic data supported the significance of Lake Melville from other regions, whereas evolutionary significance of all three groups was further supported by life‐history differences, climate‐linked differences, as well as ecological differences. A map of salmon‐bearing rivers in these three proposed DUs are shown in Figure S4 to highlight the boundaries between these proposed DUs.

Finally, while we focus on Labrador DUs here, the evolutionary significance of these DUs must also be evaluated against any neighboring DUs (see Figure 1). For Labrador, adjacent DUs to the south and north (Quebec Eastern North Shore DU and Nunavik DU, respectively) were evaluated and their significance has been previously supported in prior evaluation of COSEWIC DUs for Atlantic salmon (COSEWIC, 2010b). Although we do not go into details on these analyses here, additional details are summarized in Table S7 (COSEWIC, in prep; Lehnert et al., 2023).

## 4 DISCUSSION

Large genomic datasets are providing new opportunities to incorporate these data into conservation planning in ways that will improve the ability to accurately identify conservation units within species. Our framework presented here provides a weight of evidence approach for identifying conservation units in Canada that incorporates genetic and genomic datasets combined with complementary data types. The use of a weight of evidence approach to confirm the validity of a DU after discreteness has been established should provide robustness to over splitting (Coates et al., 2018). In this study, we demonstrate how our framework can delineate new COSEWIC DUs of anadromous Atlantic salmon within a northern region of Canada that was previously data limited. While the original single Labrador DU defined by COSEWIC in 2010 represented an extensive geographic area extending over 1300 km of coastline (COSEWIC, 2010b), our framework incorporating large genetic, genomic, and complementary datasets have revealed that there are actually three DUs within this region. While evidence of three genetic groups was previously reported in Labrador (Bradbury et al., 2018; Bradbury et al., 2021), these studies did not investigate the evolutionary significance of these units required to meet full criteria of a DU. Here, our weight of evidence approach enables the identification of DUs that better align with COSEWIC's definitions, while at the same time providing more repeatability and thus stability in DU structure. Further, given that the definition of DUs aligns with that of evolutionarily significant units (ESUs) (Waples, 1991), our approach is broadly applicable for informing conservation units outside of Canada.

The framework presented here acknowledges the hierarchical genetic structure that characterizes many species (Janes et al., 2017). The approach begins with identifying higher levels genetic groups, which can be accomplished through unsupervised analyses, and such approaches have been used previously in Atlantic salmon (Moore et al., 2014) and in recent studies aiming to characterize conservation units in other species (Forester et al., 2022; Xuereb et al., 2022). For example, in coho salmon (*Oncorhynchus kisutch*; Waulbaum, 1792), broad‐scale population structure across conservation units was evaluated from SNP data using PCA and by estimating individual ancestry coefficients (Xuereb et al., 2022), and similar approaches were applied in the Columbia spotted frog (*Rana luteiventris*, Thompson, 1913) to evaluate discreteness among units using neutral genomic data (Forester et al., 2022). After identifying higher level genetic group, our approach next focused on evaluating whether there was evidence of genetic discreteness at a finer spatial scale. In our application within Labrador, three genetically discrete groups were supported by both microsatellite and SNP datasets, with different datasets supporting different axes of primary structure. These genetic datasets also supported further evidence of genetic structure in Labrador beyond three genetic groups (Bradbury et al., 2018), although in many cases, support for evolutionary significance at such a fine scale is often lacking in Atlantic salmon (see Lehnert et al., 2023), either due to a real absence of adaptive differences at such scales or sparse data. For example, given the remote location of the Labrador DUs, data on life history were generally limited, as less than 5% of salmon‐bearing rivers are annually monitored across Labrador. Nonetheless, available syntheses of life‐history data supported differences among the three discrete genetic groups (see Table 2). Further, as we demonstrate, there are multiple data sources that can be leveraged to inform evolutionary significance to support COSEWIC's criteria of a DU, some of which can provide fine‐scale data.

In addition to life‐history data, we also evaluated genomic and climatic data to infer evolutionary significance, where in both cases data were available for a larger proportion of rivers relative to life‐history data. Genomic data provided support for putative adaptive differences between Lake Melville and coastal regions of Labrador based on all lines evidence provided in our framework (see Table 1), including frequency differences in a structural variant and genotype–environment associations. Further, our PCA revealed that outlier loci contributing to differences were found within/near genes that were associated with overrepresented biological processes, particularly “fatty acid homeostasis,” which also supports one line of evidence in our framework for putative genomic‐based adaptation (see Table 1). In addition, evaluation of climatic data supported evidence of significant climate‐linked differences likely to give rise to local adaptation between all three major genetic groups. Using redundancy analysis (RDA), we found differences in temperatures and precipitation, as well as their variability, that significantly separated all three regions of Labrador. Ecological differences further supported differences among the three genetic groups based on differences in salmonid community structure and river gradients (DFO & MNRF, 2009), and while not directly included in our decision tree framework, these differences helped further corroborate the delineation of the three DUs based on other data types. While costly targeted genetic and genomic sampling has been ongoing to accumulate the information needed to resolve differences in this region, climatic data were compiled from publicly available sources (Fick & Hijmans, 2017), highlighting the utility of such open databases for supporting evolutionary significance in other species. Overall, our analyses highlight the importance of incorporating multiple data types, especially when sampling coverage varies. Other criteria that have been used to identify evolutionary significance that could be incorporated into a weight of evidence approach include occupation of different biogeographic regions or ecozones, differences in intrinsic population growth rates, phylogenetic divergence, and differences in migration and movement strategies (COSEWIC, 2010a, 2011, 2017; Mee et al., 2015).

Although Atlantic salmon represent an extraordinary example where extensive amounts of genetic and genomic data that can inform conservation units are available, we expect that such large datasets will become increasingly common over the next decade for many species, as genomic resources become more widely accessible (Blanchet et al., 2017; Leigh et al., 2021). Already genetic datasets have been used to inform genetic discreteness or isolation of conservation units in recent decades (COSEWIC, 2010b; Mee et al., 2015; Walter et al., 2022). However, genomic datasets are only starting to be used to inform on the question of evolutionary significance (COSEWIC, 2010a; Forester et al., 2022; Waples et al., 2022; Xuereb et al., 2022). Genomic data combined with complementary datasets (e.g., dispersal and landscape data) were recently used to identify evolutionarily significant units (ESUs) and management units in a desert frog species (Forester et al., 2022). In this example, Forester et al. (2022) found evidence of genetic isolation (discreteness) among sampling locations; however, genetic–environment association analysis did not support adaptive divergence (evolutionary significance) required to meet their full criteria of ESUs. In coho salmon, current conservation units were evaluated using genomic data, where both neutral and adaptive genomic differences were assessed (Xuereb et al., 2022). In this case, consistent patterns of population structure were supported by both neutral and adaptive loci, as is the case in Atlantic salmon (Moore et al., 2014), and this genetic structure generally corresponded to many of the current conservation unit boundaries with some exceptions (Xuereb et al., 2022). In Atlantic cod (*Gadus morhua*, L.), genomic data have been used to support evolutionarily significant differences among several DUs based on genetic–environment associations linked to ocean temperature (Bradbury et al., 2010; COSEWIC, 2010a). Recent genome sequencing of caribou (*Rangifer tarandus*, L.) has revealed that current DU structure warrants changes, as data revealed evidence of parallel evolution of ecotypes, complex demographic histories, and patterns of introgression among DUs (Cavedon et al., 2022; Taylor et al., 2020).

In the examples above, many genomic markers that are distributed genome‐wide are used to inform conservation units because adaptive differences, such as environmental adaptation, are often polygenic in nature (Forester et al., 2022; Xuereb et al., 2022). However, recent work on Pacific salmon has highlighted how small genomic regions can have large effects on phenotype, and this has stimulated considerable debate about how to apply this new information into conservation units (Waples et al., 2022). In Pacific salmon, one specific genomic region of large effect has been identified as driving adaptive differences in run timing in both Chinook salmon (*O. tshawytscha*; Walbaum 1792) and steelhead (*O. mykiss*; Walbaum, 1792) (Prince et al., 2017). The genomic region, localized near the *GREB1L* gene, influences whether individuals migrate to freshwater early or late in the season, and also drives other differences in phenotype (e.g., flesh quality, fat content) (Prince et al., 2017; Thompson et al., 2019; Waples et al., 2022; Waples & Lindley, 2018). Although early and late run individuals only differ at one locus, several arguments have been made both for and against splitting these individuals into separate conservation units (reviewed in Waples et al., 2022). However, in several cases, early and late run individuals interbreed (Waples et al., 2022), and thus under COSEWIC's definition, separate DUs would not be supported as the criteria of genetic discreteness would not be met. We acknowledge these types of large effect loci in our framework approach (see Table 1), and for example, a gene of large effect (*vgll3*) has been identified that influences age‐at‐maturity in Atlantic salmon, accounting for almost 40% of variation in phenotype (Barson et al., 2015; Sinclair‐Waters et al., 2020). In our framework, we recognize that this gene can be important for differentiating salmon at a larger geographic scale. For example, large differences in the frequency of the *vgll3* “early” allele could be associated with large differences in the proportion of early maturing (i.e., one‐sea‐winter, 1‐SW) salmon between two DUs, supporting evolutionarily significant differences. However, in our framework (and under COSEWIC), we would not consider interbreeding individuals within rivers with different genotypes (and thus different age‐at‐maturity) as different DUs because discreteness is not met in this scenario. This instead represents the diversity captured within the conservation units (Funk et al., 2012; Waples et al., 2022) that warrants protecting as a whole. Further, it is worth noting that while *vgll3* underpins differences in age‐at‐maturity in European populations of Atlantic salmon, recent work suggests that this locus influences age‐at‐maturity in some populations in Canada but not others (Kess et al., 2022). Therefore, caution is warranted when making inferences about the genetic basis of ecologically and evolutionarily relevant traits to inform conservation units if the locus of interest has not been directly investigated in the populations. Overall, we agree with advice that the average pattern of genome‐wide variation should be used to inform conservation units, particularly for discreteness, rather than a single genomic region (Ford et al., 2020).

In addition to the genomic approaches used in our framework, there are several different ways in which genomic data can be used to examine putative adaptive‐based differences. For example, studies using genotype–environment associations (GEA), such as RDA or random forest analysis (Forester et al., 2018), can provide genomic‐based evidence of adaptation that could be used to delineate conservation units based on environmental adaptation (Forester et al., 2022; Xuereb et al., 2022). Similarly, genome‐wide association studies (GWAS) can identify candidate genes that underpin adaptive phenotypes (Barson et al., 2015; Lehnert, Christensen, et al., 2019), and as discussed above, identifying large‐scale differences in allele frequencies of such genes can also inform on differences in adaptive traits among conservation units.

In our approach, we also capture additional sources of variation in the genome beyond SNPs, such as structural variants (Mérot et al., 2020), which were used to delineate conservation units in Labrador, and have also been used in other regions of Canada including southern Newfoundland (Lehnert, Bentzen, et al., 2019; Lehnert et al., 2023; Watson et al., 2022). With advances in sequencing technology continually expanding, organizations such as COSEWIC must be prepared to integrate additional sources of adaptative variation into conservation planning, such as genome structure, transcriptomic, and methylation data (Anastasiadi et al., 2021; Meröndun et al., 2019; Mérot et al., 2020; Verta & Jacobs, 2022). Although in the case of transcriptomic and methylation data, signals can be influenced by short‐term environmental factors (Anastasiadi et al., 2021; Verta & Jacobs, 2022) and can only be informative for significance if a heritable basis is confirmed, such as through common‐garden experiments. While molecular datasets provide insight into the potential basis of isolation and putative adaptive divergence, and thus could be used to fully address COSEWIC's criteria of DUs, we suggest that genomic data should not be used as standalone support for DUs, and corroborating data are needed to develop robust DU boundaries (see also Coates et al., 2018). We recommend relying on multiple data types using a multifaceted approach (as we present here) to provide strong support for DUs, specifically for evolutionary significance, that ensure the stability of DU structure into the future. Changes to DU structure can influence the status, conservation strategies, and recovery of the units (Taylor et al., 2020), and thus stability in the knowledge of DU structure is important for managers and recovery efforts. While new data always have the possibility to lead to changes, using a weight of evidence approach can help ensure the most appropriate DUs are identified. Finally, while climate change may influence species distribution resulting in northward shifts (Reist et al., 2006), we would not expect this to change DU structure, but instead may result in the formation of new DUs in habitats that are not currently occupied by the species.

In conclusion, our work highlights how genetic, but more so genomic data, have the capacity to transform our ability to address criteria of conservation units, such as those defined under COSEWIC. Our framework has been applied to anadromous Atlantic salmon populations across eastern Canada, and in Labrador has resulted in the subdivision of a previously data limited single, large DU. These changes may improve conservation and management of Labrador populations and help protect unique biodiversity in the species, as currently the three DUs are experiencing different trends in abundance over time (COSEWIC, in prep). Differences in trends indicate that these regions may be subjected to different threats, and thus conservation and management needs of these units may differ. In addition, while our results for all regions of eastern Canada are not fully presented here, the application of this framework has resulted in substantial changes to the DU structure of this species, increasing the number of DUs by four compared to the previous assessment, and also resulted in boundary changes, subdivisions, and mergers of previously recognized DUs (Figure S9) (COSEWIC, in prep; Lehnert et al., 2023). These changes result in units that now better align with the definition of a DU and have been approved by COSEWIC. Our work on Atlantic salmon represents a new and higher standard for the future characterization of DUs for wildlife species in Canada. Although this framework was designed to meet criteria of COSEWIC DUs, our approach is widely applicable for informing conservation units in other areas, provided the parallels in the definition of these units (Fraser & Bernatchez, 2001; Waples, 1991).

## CONFLICT OF INTEREST STATEMENT

We declare no conflict of interest.

## Supporting information


Figure S1.

## Data Availability

All scripts for running analyses described here are available at https://github.com/SarahLehnert/Salmon_DUs. Data used here were accessed from previous publications, and these included the 101 microsatellite dataset (Sylvester et al., 2018), 96 SNP dataset (Bradbury et al., 2021), and 220K SNP dataset (Lehnert et al., 2023). The genomic dataset used for Labrador is available on Dryad at https://doi.org/10.5061/dryad.6wwpzgn4h.

## References

[eva13587-bib-0001] Alexa, A. , & Rahnenfuhrer, J. (2016). topGO: Enrichment analysis for Gene Ontology. R package version 2.28.0.

[eva13587-bib-0002] Alexander, D. H. , & Lange, K. (2011). Enhancements to the ADMIXTURE algorithm for individual ancestry estimation. BMC Bioinformatics, 12, 1–6. 21682921 10.1186/1471-2105-12-246 PMC3146885

[eva13587-bib-0003] Anastasiadi, D. , Venney, C. J. , Bernatchez, L. , & Wellenreuther, M. (2021). Epigenetic inheritance and reproductive mode in plants and animals. Trends in Ecology & Evolution, 36, 1124–1140. 34489118 10.1016/j.tree.2021.08.006

[eva13587-bib-0004] Barson, N. J. , Aykanat, T. , Hindar, K. , Baranski, M. , Bolstad, G. H. , Fiske, P. , Jacq, C. , Jensen, A. J. , Johnston, S. E. , Karlsson, S. , Kent, M. P. , Niemelä, E. , Nome, T. , Næsje, T. F. , Orell, P. , Romakkaniemi, A. , Sægrov, H. , Urdal, K. , Erkinaro, J. , … Primmer, C. R. (2015). Sex‐dependent dominance at a single locus maintains variation in age at maturity in salmon. Nature, 528, 405–408. 26536110 10.1038/nature16062

[eva13587-bib-0005] Besnier, F. , & Glover, K. A. (2013). ParallelStructure: A R package to distribute parallel runs of the population genetics program STRUCTURE on multi‐core computers. PLoS One, 8, e70651. 23923012 10.1371/journal.pone.0070651 PMC3726640

[eva13587-bib-0006] Blanchet, S. , Prunier, J. G. , & De Kort, H. (2017). Time to go bigger: Emerging patterns in macrogenetics. Trends in Genetics, 33, 579–580. 28720482 10.1016/j.tig.2017.06.007

[eva13587-bib-0007] Bradbury, I. R. , Hamilton, L. C. , Robertson, M. J. , Bourgeois, C. E. , Mansour, A. , & Dempson, J. B. (2014). Landscape structure and climatic variation determine Atlantic salmon genetic connectivity in the Northwest Atlantic. Canadian Journal of Fisheries and Aquatic Sciences, 71, 246–258.

[eva13587-bib-0008] Bradbury, I. R. , Hubert, S. , Higgins, B. , Borza, T. , Bowman, S. , Paterson, I. G. , Snelgrove, P. V. , Morris, C. J. , Gregory, R. S. , & Hardie, D. C. (2010). Parallel adaptive evolution of Atlantic cod on both sides of the Atlantic Ocean in response to temperature. Proceedings of the Royal Society B: Biological Sciences, 277, 3725–3734. 10.1098/rspb.2010.0985 PMC2992707 20591865

[eva13587-bib-0009] Bradbury, I. R. , Lehnert, S. J. , Messmer, A. M. , Duffy, S. J. , Verspoor, E. , Kess, T. , Gilbey, J. , Wennevik, V. , Robertson, M. J. , Chaput, G. , Sheehan, T. F. , Bentzen, P. , Dempson, J. B. , & Reddin, D. G. (2021). Range‐wide genetic assignment confirms long‐distance oceanic migration in Atlantic salmon over half a century. ICES Journal of Marine Science, 78, 1434–1443.

[eva13587-bib-0010] Bradbury, I. R. , Wringe, B. F. , Watson, B. , Paterson, I. , Horne, J. , Beiko, R. , Lehnert, S. J. , Clément, M. , Anderson, E. C. , Jeffery, N. W. , Duffy, S. , Sylvester, E. , Martha, R. , & Bentzen, P. (2018). Genotyping‐by‐sequencing of genome‐wide microsatellite loci reveals fine‐scale harvest composition in a coastal Atlantic salmon fishery. Evolutionary Applications, 11, 918–930. 29928300 10.1111/eva.12606 PMC5999200

[eva13587-bib-0011] Cauwelier, E. , Gilbey, J. , Sampayo, J. , Stradmeyer, L. , & Middlemas, S. J. (2018). Identification of a single genomic region associated with seasonal river return timing in adult Scottish Atlantic salmon (*Salmo salar* L.) identified using a genome‐wide association study. Canadian Journal of Fisheries and Aquatic Sciences, 75, 1427–1435.

[eva13587-bib-0012] Cavedon, M. , Poissant, J. , VonHoldt, B. , Michalak, A. , Hegel, T. , Heppenheimer, E. , Hervieux, D. , Neufeld, L. , Polfus, J. L. , & Schwantje, H. (2022). Population structure of threatened caribou in western Canada inferred from genome‐wide SNP data. Conservation Genetics, 23, 1089–1103.

[eva13587-bib-0013] Ceballos, G. , Ehrlich, P. R. , & Raven, P. H. (2020). Vertebrates on the brink as indicators of biological annihilation and the sixth mass extinction. Proceedings of the National Academy of Sciences, 117, 13596–13602. 10.1073/pnas.1922686117 PMC7306750 32482862

[eva13587-bib-0014] Chaput, G. , Dempson, J. B. , Caron, F. , Jones, R. , & Gibson, J. (2006). A synthesis of life history characteristics and stock grouping of Atlantic salmon (Salmo salar L.) in eastern Canada . Canadian Science Advisory Secretariat 2006/015.

[eva13587-bib-0015] Coates, D. J. , Byrne, M. , & Moritz, C. (2018). Genetic diversity and conservation units: Dealing with the species‐population continuum in the age of genomics. Frontiers in Ecology and Evolution, 6, 165.

[eva13587-bib-0016] COSEWIC . (2010a). COSEWIC assessment and status report on the Atlantic cod Gadus morhua in Canada. Committee on the Status of Endangered Wildlife in Canada, Ottawa. xiii+105 pp.

[eva13587-bib-0017] COSEWIC . (2010b). COSEWIC assessment and status report on the Atlantic Salmon Salmo salar (Nunavik population, Labrador population, Northeast Newfoundland population, South Newfoundland population, Southwest Newfoundland population, Northwest Newfoundland population, Quebec eastern north shore population, Quebec Western north shore population, Anticosti Island population, inner St. Lawrence population, Lake Ontario population, Gaspé‐southern gulf of St. Lawrence population, eastern cape Breton population, Nova Scotia southern upland population, Inner Bay of Fundy population, Outer Bay of Fundy population) in Canada. Committee on the status of endangered wildlife in Canada. Committee on the Status of Endangered Wildlife in Canada, Ottawa. xlvii+136 pp.

[eva13587-bib-0018] COSEWIC . (2011). Designatable units for caribou (Rangifer tarandus) in Canada. Committee on the Status of Endangered Wildlife in Canada, Ottawa. 88p.

[eva13587-bib-0019] COSEWIC . (2017). COSEWIC assessment and status report on the sockeye Salmon Oncorhynchus nerka, 24 Designatable units in the Fraser River Drainage Basin, in Canada. Committee on the Status of Endangered Wildlife in Canada, Ottawa. xli+179 pp.

[eva13587-bib-0020] COSEWIC . (2020). COSEWIC guidelines for recognizing designatable units. https://cosewic.ca/index.php/en‐ca/reports/preparing‐status‐reports/guidelines‐recognizing‐designatable‐units.html

[eva13587-bib-0021] COSEWIC . (in prep). COSEWIC status report on the Atlantic Salmon (*Salmo salar*) in Canada. Committee on the Status of Endangered Wildlife in Canada, Ottawa.

[eva13587-bib-0022] Des Roches, S. , Pendleton, L. H. , Shapiro, B. , & Palkovacs, E. P. (2021). Conserving intraspecific variation for nature's contributions to people. Nature Ecology & Evolution, 5, 574–582. 33649544 10.1038/s41559-021-01403-5

[eva13587-bib-0023] Des Roches, S. , Post, D. M. , Turley, N. E. , Bailey, J. K. , Hendry, A. P. , Kinnison, M. T. , Schweitzer, J. A. , & Palkovacs, E. P. (2018). The ecological importance of intraspecific variation. Nature Ecology & Evolution, 2, 57–64. 29203921 10.1038/s41559-017-0402-5

[eva13587-bib-0024] Desforges, J. E. , Clarke, J. , Harmsen, E. J. , Jardine, A. M. , Robichaud, J. A. , Serré, S. , Chakrabarty, P. , Bennett, J. R. , Hanna, D. E. , & Smol, J. P. (2022). The alarming state of freshwater biodiversity in Canada. Canadian Journal of Fisheries and Aquatic Sciences, 79, 352–365.

[eva13587-bib-0025] DFO & MNRF . (2009). Conservation status report, Atlantic salmon in Atlantic Canada and Québec: Part I–species information . Canadian Manuscript Report of Fisheries and Aquatic Sciences No. 2861.

[eva13587-bib-0026] Dionne, M. , Miller, K. M. , Dodson, J. J. , Caron, F. , & Bernatchez, L. (2007). Clinal variation in MHC diversity with temperature: Evidence for the role of host–pathogen interaction on local adaptation in Atlantic salmon. Evolution, 61, 2154–2164. 17767587 10.1111/j.1558-5646.2007.00178.x

[eva13587-bib-0027] Earl, D. A. , & VonHoldt, B. (2012). STRUCTURE HARVESTER: A website and program for visualizing STRUCTURE output and implementing the Evanno method. Conservation Genetics Resources, 4, 359–361.

[eva13587-bib-0028] Evanno, G. , Regnaut, S. , & Goudet, J. (2005). Detecting the number of clusters of individuals using the software STRUCTURE: A simulation study. Molecular Ecology, 14, 2611–2620. 15969739 10.1111/j.1365-294X.2005.02553.x

[eva13587-bib-0029] Exposito‐Alonso, M. (2017). rbioclim: improved getData function from the raster R package to interact with past, present and future climate data from worldclim.org. http://github.com/MoisesExpositoAlonso/rbioclim

[eva13587-bib-0030] Fick, S. E. , & Hijmans, R. J. (2017). WorldClim 2: New 1‐km spatial resolution climate surfaces for global land areas. International Journal of Climatology, 37, 4302–4315.

[eva13587-bib-0031] Ford, M. D. , Nichols, K. M. , Waples, R. S. , Anderson, E. C. , Kardos, M. , Koch, I. , McKinney, G. , Miller, M. R. , Myers, J. , Naish, K. , Narum, S. , O'Malley, K. G. , Pearse, D. , Seamons, T. , Spidle, A. , Swanson, P. , Thompson, T. Q. , Warheit, K. , & Willis, S. (2020). Reviewing and synthesizing the state of the science regarding associations between adult run timing and specific genotypes in Chinook salmon and steelhead: Report of a workshop held in Seattle, Washington, 27–28 February 2020. U.S. Department of Commerce, NOAA Processed Report, NMF‐NWFSC‐PR‐2020‐06.

[eva13587-bib-0032] Forester, B. , & Lama, T. (2022). The role of genomics in the future of ESA decision‐making. EcoEvoRxiv.

[eva13587-bib-0033] Forester, B. R. , Lasky, J. R. , Wagner, H. H. , & Urban, D. L. (2018). Comparing methods for detecting multilocus adaptation with multivariate genotype–environment associations. Molecular Ecology, 27, 2215–2233. 29633402 10.1111/mec.14584

[eva13587-bib-0034] Forester, B. R. , Murphy, M. , Mellison, C. , Petersen, J. , Pilliod, D. S. , Van Horne, R. , Harvey, J. , & Funk, W. C. (2022). Genomics‐informed delineation of conservation units in a desert amphibian. Molecular Ecology, 31, 5249–5269. 35976166 10.1111/mec.16660 PMC9804278

[eva13587-bib-0035] Fraser, D. J. , & Bernatchez, L. (2001). Adaptive evolutionary conservation: Towards a unified concept for defining conservation units. Molecular Ecology, 10, 2741–2752. 11903888

[eva13587-bib-0036] Frichot, E. , & François, O. (2015). LEA: An R package for landscape and ecological association studies. Methods in Ecology and Evolution, 6, 925–929.

[eva13587-bib-0037] Funk, W. C. , McKay, J. K. , Hohenlohe, P. A. , & Allendorf, F. W. (2012). Harnessing genomics for delineating conservation units. Trends in Ecology & Evolution, 27, 489–496. 22727017 10.1016/j.tree.2012.05.012 PMC4185076

[eva13587-bib-0038] Gutierrez, A. P. , Yáñez, J. M. , Fukui, S. , Swift, B. , & Davidson, W. S. (2015). Genome‐wide association study (GWAS) for growth rate and age at sexual maturation in Atlantic salmon (*Salmo salar*). PLoS One, 10, e0119730. 25757012 10.1371/journal.pone.0119730 PMC4355585

[eva13587-bib-0039] He, X. , Chaganti, S. R. , & Heath, D. D. (2018). Population‐specific responses to interspecific competition in the gut microbiota of two Atlantic Salmon (*Salmo salar*) populations. Microbial Ecology, 75, 140–151. 28714057 10.1007/s00248-017-1035-6

[eva13587-bib-0040] Helgeland, H. , Sodeland, M. , Zoric, N. , Torgersen, J. S. , Grammes, F. , von Lintig, J. , Moen, T. , Kjøglum, S. , Lien, S. , & Våge, D. I. (2019). Genomic and functional gene studies suggest a key role of beta‐carotene oxygenase 1 like (*bco1l*) gene in salmon flesh color. Scientific Reports, 9, 1–12. 31882713 10.1038/s41598-019-56438-3 PMC6934663

[eva13587-bib-0041] Hutchings, J. A. , & Jones, M. E. (1998). Life history variation and growth rate thresholds for maturity in Atlantic salmon, Salmo salar. Canadian Journal of Fisheries and Aquatic Sciences, 55, 22–47.

[eva13587-bib-0042] Janes, J. K. , Miller, J. M. , Dupuis, J. R. , Malenfant, R. M. , Gorrell, J. C. , Cullingham, C. I. , & Andrew, R. L. (2017). The K = 2 conundrum. Molecular Ecology, 26, 3594–3602. 28544181 10.1111/mec.14187

[eva13587-bib-0043] Jeffery, N. W. , Stanley, R. R. , Wringe, B. F. , Guijarro‐Sabaniel, J. , Bourret, V. , Bernatchez, L. , Bentzen, P. , Beiko, R. G. , Gilbey, J. , Clément, M. , & Bradbury, I. R. (2017). Range‐wide parallel climate‐associated genomic clines in Atlantic salmon. Royal Society Open Science, 4, 171394. 29291123 10.1098/rsos.171394 PMC5717698

[eva13587-bib-0044] Jeffery, N. W. , Wringe, B. F. , McBride, M. C. , Hamilton, L. C. , Stanley, R. R. E. , Bernatchez, L. , Clement, M. , Gilbey, J. , Sheehan, T. F. , Bentzen, P. , & Bradbury, I. R. (2018). Range‐wide regional assignment of Atlantic salmon (*Salmo salar*) using genome wide single‐nucleotide polymorphisms. Fisheries Research, 206, 163–175.

[eva13587-bib-0045] Jombart, T. (2008). Adegenet: A R package for the multivariate analysis of genetic markers. Bioinformatics, 24, 1403–1405. 18397895 10.1093/bioinformatics/btn129

[eva13587-bib-0046] Kelly, N. , Burke, C. , Lancaster, D. , Lehnert, S. J. , Loughlin, K. , Van Leeuwen, T. , Dempson, B. , Poole, R. , Robertson, M. , & Bradbury, I. (in prep). Updated information on Atlantic Salmon (Salmo salar) populations in Labrador of relevance to the COSEWIC status report . DFO Canadian Science Advisory Secretariat, Res. Doc. 2021/XXX.

[eva13587-bib-0047] Kess, T. , Lehnert, S. J. , Bentzen, P. , Duffy, S. , Messmer, A. , Dempson, J. B. , Newport, J. , Whidden, C. , Robertson, M. J. , & Chaput, G. (2022). Parallel genomic basis of age at maturity across spatial scales in Atlantic Salmon. Biorxiv. 10.1002/ece3.11068 PMC10995719 38584771

[eva13587-bib-0048] King, T. , Kalinowski, S. T. , Schill, W. , Spidle, A. , & Lubinski, B. (2001). Population structure of Atlantic salmon (*Salmo salar* L.): A range‐wide perspective from microsatellite DNA variation. Molecular Ecology, 10, 807–821. 11348491 10.1046/j.1365-294x.2001.01231.x

[eva13587-bib-0049] Kjærner‐Semb, E. , Ayllon, F. , Furmanek, T. , Wennevik, V. , Dahle, G. , Niemelä, E. , Ozerov, M. , Vähä, J.‐P. , Glover, K. A. , & Rubin, C. J. (2016). Atlantic salmon populations reveal adaptive divergence of immune related genes‐a duplicated genome under selection. BMC Genomics, 17, 610. 27515098 10.1186/s12864-016-2867-z PMC4982270

[eva13587-bib-0050] Klemetsen, A. , Amundsen, P. A. , Dempson, J. B. , Jonsson, B. , Jonsson, N. , O'Connell, M. F. , & Mortensen, E. (2003). Atlantic salmon *Salmo salar* L., brown trout *Salmo trutta* L. and Arctic charr *Salvelinus alpinus* (L.): A review of aspects of their life histories. Ecology of Freshwater Fish, 12, 1–59.

[eva13587-bib-0051] Kopelman, N. M. , Mayzel, J. , Jakobsson, M. , Rosenberg, N. A. , & Mayrose, I. (2015). Clumpak: A program for identifying clustering modes and packaging population structure inferences across K. Molecular Ecology Resources, 15, 1179–1191. 25684545 10.1111/1755-0998.12387 PMC4534335

[eva13587-bib-0052] Layton, K. K. S. , Snelgrove, P. V. R. , Dempson, J. B. , Kess, T. , Lehnert, S. J. , Bentzen, P. , Duffy, S. J. , Messmer, A. M. , Stanley, R. R. E. , DiBacco, C. , Salisbury, S. J. , Ruzzante, D. E. , Nugent, C. M. , Ferguson, M. M. , Leong, J. S. , Koop, B. F. , & Bradbury, I. R. (2021). Genomic evidence of past and future climate‐linked loss in a migratory Arctic fish. Nature Climate Change, 11, 158–165.

[eva13587-bib-0053] Lehnert, S. J. , Bentzen, P. , Kess, T. , Lien, S. , Horne, J. B. , Clement, M. , & Bradbury, I. R. (2019). Chromosome polymorphisms track trans‐Atlantic divergence and secondary contact in Atlantic salmon. Molecular Ecology, 28, 2074–2087. 30825352 10.1111/mec.15065

[eva13587-bib-0054] Lehnert, S. J. , Bradbury, I. R. , April, J. , Wringe, B. F. , Van Wyngaarden, M. , & Bentzen, P. (2023). Pre‐COSEWIC Review of Anadromous Atlantic Salmon (Salmo salar) in Canada, Part 1: Designatable Units . DFO Canadian Science Advisory Secretariat, Res. Doc. 2023/026.

[eva13587-bib-0055] Lehnert, S. J. , Christensen, K. A. , Vandersteen, W. E. , Sakhrani, D. , Pitcher, T. E. , Heath, J. W. , Koop, B. F. , Heath, D. D. , & Devlin, R. H. (2019). Carotenoid pigmentation in salmon: Variation in expression at BCO2‐l locus controls a key fitness trait affecting red coloration. Proceedings of the Royal Society B, 286, 20191588. 31615356 10.1098/rspb.2019.1588 PMC6834058

[eva13587-bib-0056] Lehnert, S. J. , Kess, T. , Bentzen, P. , Clement, M. , & Bradbury, I. R. (2020). Divergent and linked selection shape patterns of genomic differentiation between European and North American Atlantic salmon (*Salmo salar*). Molecular Ecology, 29, 2160–2175. 32432380 10.1111/mec.15480

[eva13587-bib-0057] Lehnert, S. J. , Kess, T. , Bentzen, P. , Kent, M. P. , Lien, S. , Gilbey, J. , Clement, M. , Jeffery, N. W. , Waples, R. S. , & Bradbury, I. R. (2019). Genomic signatures and correlates of widespread population declines in salmon. Nature Communications, 10, 2996. 10.1038/s41467-019-10972-w PMC6611788 31278264

[eva13587-bib-0058] Leigh, D. M. , van Rees, C. B. , Millette, K. L. , Breed, M. F. , Schmidt, C. , Bertola, L. D. , Hand, B. K. , Hunter, M. E. , Jensen, E. L. , & Kershaw, F. (2021). Opportunities and challenges of macrogenetic studies. Nature Reviews Genetics, 22, 791–807. 10.1038/s41576-021-00394-0 34408318

[eva13587-bib-0059] Lien, S. , Koop, B. F. , Sandve, S. R. , Miller, J. R. , Kent, M. P. , Nome, T. , Hvidsten, T. R. , Leong, J. S. , Minkley, D. R. , & Zimin, A. (2016). The Atlantic salmon genome provides insights into rediploidization. Nature, 533, 200–205. 27088604 10.1038/nature17164 PMC8127823

[eva13587-bib-0060] Luu, K. , Bazin, E. , & Blum, M. G. (2017). Pcadapt: An R package to perform genome scans for selection based on principal component analysis. Molecular Ecology Resources, 17, 67–77. 27601374 10.1111/1755-0998.12592

[eva13587-bib-0061] Mee, J. A. , Bernatchez, L. , Reist, J. D. , Rogers, S. M. , & Taylor, E. B. (2015). Identifying designatable units for intraspecific conservation prioritization: A hierarchical approach applied to the lake whitefish species complex (Coregonus spp.). Evolutionary Applications, 8, 423–441. 26029257 10.1111/eva.12247 PMC4430767

[eva13587-bib-0062] Meröndun, J. , Murray, D. L. , & Shafer, A. B. (2019). Genome‐scale sampling suggests cryptic epigenetic structuring and insular divergence in Canada lynx. Molecular Ecology, 28, 3186–3196. 31087722 10.1111/mec.15131

[eva13587-bib-0063] Mérot, C. , Oomen, R. A. , Tigano, A. , & Wellenreuther, M. (2020). A roadmap for understanding the evolutionary significance of structural genomic variation. Trends in Ecology & Evolution, 35, 561–572. 32521241 10.1016/j.tree.2020.03.002

[eva13587-bib-0064] Metcalfe, N. , & Thorpe, J. (1990). Determinants of geographical variation in the age of seaward‐migrating salmon, *Salmo salar* . The Journal of Animal Ecology, 59, 135–145.

[eva13587-bib-0065] Miettinen, A. , Palm, S. , Dannewitz, J. , Lind, E. , Primmer, C. R. , Romakkaniemi, A. , Östergren, J. , & Pritchard, V. L. (2021). A large wild salmon stock shows genetic and life history differentiation within, but not between, rivers. Conservation Genetics, 22, 35–51.

[eva13587-bib-0066] Moore, J. S. , Bourret, V. , Dionne, M. , Bradbury, I. , O'Reilly, P. , Kent, M. , Chaput, G. , & Bernatchez, L. (2014). Conservation genomics of anadromous Atlantic salmon across its north American range: Outlier loci identify the same patterns of population structure as neutral loci. Molecular Ecology, 23, 5680–5697. 25327895 10.1111/mec.12972

[eva13587-bib-0067] Oksanen, J. , Blanchet, G. F. , Friendly, M. , Kindt, R. , Legendre, P. , McGlinn, D. , Minchin, P. , O'Hara, R. B. , Simpson, G. L. , Solymos, P. , Stevens, M. H. H. , Szoecs, E. , & Wagner, H. (2017). vegan: community ecology package R package version 2.4‐3. https://CRAN.R‐project.org/package=vegan

[eva13587-bib-0068] Páez, D. , Brisson‐Bonenfant, C. , Rossignol, O. , Guderley, H. E. , Bernatchez, L. , & Dodson, J. J. (2011). Alternative developmental pathways and the propensity to migrate: A case study in the Atlantic salmon. Journal of Evolutionary Biology, 24, 245–255. 21044203 10.1111/j.1420-9101.2010.02159.x

[eva13587-bib-0069] Pearse, D. E. , Barson, N. J. , Nome, T. , Gao, G. , Campbell, M. A. , Abadía‐Cardoso, A. , Anderson, E. C. , Rundio, D. E. , Williams, T. H. , Naish, K. A. , Moen, T. , Liu, S. , Kent, M. , Minkley, D. R. , Rondeau, E. B. , Brieuc, M. S. O. , Sandve, S. R. , Miller, M. R. , Cedillo, L. , … Lien, S. (2019). Sex‐dependent dominance maintains migration supergene in rainbow trout. Nature Ecology & Evolution, 3, 1731–1742. 31768021 10.1038/s41559-019-1044-6

[eva13587-bib-0070] Prince, D. J. , O'Rourke, S. M. , Thompson, T. Q. , Ali, O. A. , Lyman, H. S. , Saglam, I. K. , Hotaling, T. J. , Spidle, A. P. , & Miller, M. R. (2017). The evolutionary basis of premature migration in Pacific salmon highlights the utility of genomics for informing conservation. Science Advances, 3, e1603198. 28835916 10.1126/sciadv.1603198 PMC5559211

[eva13587-bib-0071] Pritchard, J. K. , Stephens, M. , & Donnelly, P. (2000). Inference of population structure using multilocus genotype data. Genetics, 155, 945–959. 10835412 10.1093/genetics/155.2.945 PMC1461096

[eva13587-bib-0072] Pritchard, V. L. , Mäkinen, H. , Vähä, J. P. , Erkinaro, J. , Orell, P. , & Primmer, C. R. (2018). Genomic signatures of fine‐scale local selection in Atlantic salmon suggest involvement of sexual maturation, energy homeostasis and immune defence‐related genes. Molecular Ecology, 27, 2560–2575. 29691916 10.1111/mec.14705

[eva13587-bib-0073] Purcell, S. , Neale, B. , Todd‐Brown, K. , Thomas, L. , Ferreira, M. A. , Bender, D. , Maller, J. , Sklar, P. , De Bakker, P. I. , & Daly, M. J. (2007). PLINK: A tool set for whole‐genome association and population‐based linkage analyses. The American Journal of Human Genetics, 81, 559–575. 17701901 10.1086/519795 PMC1950838

[eva13587-bib-0074] Quinlan, A. R. , & Hall, I. M. (2010). BEDTools: A flexible suite of utilities for comparing genomic features. Bioinformatics, 26, 841–842. 20110278 10.1093/bioinformatics/btq033 PMC2832824

[eva13587-bib-0075] Reist, J. D. , Wrona, F. J. , Prowse, T. D. , Power, M. , Dempson, J. B. , King, J. R. , & Beamish, R. J. (2006). An overview of effects of climate change on selected Arctic freshwater and anadromous fishes. AMBIO: A Journal of the Human Environment, 35, 381–387. 10.1579/0044-7447(2006)35[381:aooeoc]2.0.co;2 17256642

[eva13587-bib-0076] Rougemont, Q. , & Bernatchez, L. (2018). The demographic history of Atlantic salmon (Salmo salar) across its distribution range reconstructed from approximate Bayesian computations. Evolution, 72, 1261–1277. 29644624 10.1111/evo.13486

[eva13587-bib-0077] Ryder, O. A. (1986). Species conservation and systematics ‐ the dilemma of subspecies. Trends in Ecology & Evolution, 1, 9–10. 10.1016/0169-5347(86)90035-2 21227791

[eva13587-bib-0078] Samy, J. K. A. , Mulugeta, T. D. , Nome, T. , Sandve, S. R. , Grammes, F. , Kent, M. P. , Lien, S. , & Våge, D. I. (2017). SalmoBase: An integrated molecular data resource for salmonid species. BMC Genomics, 18, 1–5. 28651544 10.1186/s12864-017-3877-1 PMC5485693

[eva13587-bib-0079] Schaffer, W. M. , & Elson, P. F. (1975). The adaptive significance of variations in life history among local populations of Atlantic salmon in North America. Ecology, 56, 577–590.

[eva13587-bib-0080] Schindler, D. E. , Hilborn, R. , Chasco, B. , Boatright, C. P. , Quinn, T. P. , Rogers, L. A. , & Webster, M. S. (2010). Population diversity and the portfolio effect in an exploited species. Nature, 465, 609–612. 20520713 10.1038/nature09060

[eva13587-bib-0081] Sinclair‐Waters, M. , Ødegård, J. , Korsvoll, S. A. , Moen, T. , Lien, S. , Primmer, C. R. , & Barson, N. J. (2020). Beyond large‐effect loci: Large‐scale GWAS reveals a mixed large‐effect and polygenic architecture for age at maturity of Atlantic salmon. Genetics Selection Evolution, 52, 1–11. 10.1186/s12711-020-0529-8 PMC7017552 32050893

[eva13587-bib-0082] Stabell, O. B. (1984). Homing and olfaction in salmonids: A critical review with special reference to the Atlantic salmon. Biological Reviews, 59, 333–388.

[eva13587-bib-0083] Stenløkk, K. , Saitou, M. , Rud‐Johansen, L. , Nome, T. , Moser, M. , Árnyasi, M. , Kent, M. , Barson, N. J. , & Lien, S. (2022). The emergence of supergenes from inversions in Atlantic salmon. Philosophical Transactions of the Royal Society B, 377, 20210195. 10.1098/rstb.2021.0195 PMC9189505 35694753

[eva13587-bib-0084] Storey, J. D. , Bass, A. J. , Dabney, A. , & Robinson, D. (2015). qvalue: Q‐value estimation for false discovery rate control. R package version 2.0.0.

[eva13587-bib-0085] Storey, J. D. , & Tibshirani, R. (2003). Statistical significance for genomewide studies. Proceedings of the National Academy of Sciences, 100, 9440–9445. 10.1073/pnas.1530509100 PMC170937 12883005

[eva13587-bib-0086] Supek, F. , Bošnjak, M. , Škunca, N. , & Šmuc, T. (2011). REVIGO summarizes and visualizes long lists of gene ontology terms. PLoS One, 6, e21800. 21789182 10.1371/journal.pone.0021800 PMC3138752

[eva13587-bib-0087] Sylvester, E. V. , Beiko, R. G. , Bentzen, P. , Paterson, I. G. , Horne, J. B. , Watson, B. , Lehnert, S. J. , Duffy, S. , Clement, M. , Robertson, M. J. , & Bradbury, I. R. (2018). Environmental extremes drive population structure at the northern range limit of Atlantic salmon in North America. Molecular Ecology, 27, 4026–4040. 30152128 10.1111/mec.14849

[eva13587-bib-0088] Taylor, R. S. , Manseau, M. , Horn, R. L. , Keobouasone, S. , Golding, G. B. , & Wilson, P. J. (2020). The role of introgression and ecotypic parallelism in delineating intraspecific conservation units. Molecular Ecology, 29, 2793–2809. 32567754 10.1111/mec.15522 PMC7496186

[eva13587-bib-0089] Thompson, T. Q. , Bellinger, M. R. , O'Rourke, S. M. , Prince, D. J. , Stevenson, A. E. , Rodrigues, A. T. , Sloat, M. R. , Speller, C. F. , Yang, D. Y. , & Butler, V. L. (2019). Anthropogenic habitat alteration leads to rapid loss of adaptive variation and restoration potential in wild salmon populations. Proceedings of the National Academy of Sciences, 116, 177–186. 10.1073/pnas.1811559115 PMC6320526 30514813

[eva13587-bib-0090] Turner, S. D. (2014). Qqman: An R package for visualizing GWAS results using QQ and Manhattan plots. Biorxiv, 005165.

[eva13587-bib-0091] US Fish and Wildlife Service and National Oceanic and Atmospheric Administration . (2019). Recovery plan for the Gulf of Maine distinct population segment of Atlantic salmon (*Salmo salar*). 74 pp.

[eva13587-bib-0092] Vaha, J. P. , Erkinaro, J. , Niemelä, E. , & Primmer, C. R. (2007). Life‐history and habitat features influence the within‐river genetic structure of Atlantic salmon. Molecular Ecology, 16, 2638–2654. 17594436 10.1111/j.1365-294X.2007.03329.x

[eva13587-bib-0093] Verta, J.‐P. , & Jacobs, A. (2022). The role of alternative splicing in adaptation and evolution. Trends in Ecology & Evolution, 37, 299–308. 34920907 10.1016/j.tree.2021.11.010

[eva13587-bib-0094] Walter, R. P. , Venney, C. J. , Mandrak, N. E. , & Heath, D. D. (2022). Conservation implications of revised genetic structure resulting from new population discovery: The threatened eastern sand darter (*Ammocrypta pellucida*) in Canada. Journal of Fish Biology, 100, 92–98. 34643948 10.1111/jfb.14922

[eva13587-bib-0095] Waples, R. S. (1991). Pacific salmon, Oncorhynchus spp., and the definition of “species” under the endangered species act. Marine Fisheries Review, 53, 11.

[eva13587-bib-0096] Waples, R. S. , Ford, M. J. , Nichols, K. , Kardos, M. , Myers, J. , Thompson, T. Q. , Anderson, E. C. , Koch, I. J. , McKinney, G. , & Miller, M. R. (2022). Implications of large‐effect loci for conservation: A review and case study with Pacific Salmon. Journal of Heredity, 113, 121–144. 35575083 10.1093/jhered/esab069

[eva13587-bib-0097] Waples, R. S. , & Lindley, S. T. (2018). Genomics and conservation units: The genetic basis of adult migration timing in Pacific salmonids. Evolutionary Applications, 11, 1518–1526. 30344624 10.1111/eva.12687 PMC6183503

[eva13587-bib-0098] Waters, C. D. , Clemento, A. , Aykanat, T. , Garza, J. C. , Naish, K. A. , Narum, S. , & Primmer, C. R. (2021). Heterogeneous genetic basis of age at maturity in salmonid fishes. Molecular Ecology, 30, 1435–1456. 33527498 10.1111/mec.15822

[eva13587-bib-0099] Watson, K. B. , Lehnert, S. J. , Bentzen, P. , Kess, T. , Einfeldt, A. , Duffy, S. , Perriman, B. , Lien, S. , Kent, M. , & Bradbury, I. R. (2022). Environmentally associated chromosomal structural variation influences fine‐scale population structure of Atlantic Salmon (Salmo salar). Molecular Ecology, 31, 1057–1075. 34862998 10.1111/mec.16307

[eva13587-bib-0100] Wellband, K. , Mérot, C. , Linnansaari, T. , Elliott, J. , Curry, R. A. , & Bernatchez, L. (2019). Chromosomal fusion and life history‐associated genomic variation contribute to within‐river local adaptation of Atlantic salmon. Molecular Ecology, 28, 1439–1459. 30506831 10.1111/mec.14965

[eva13587-bib-0101] Wellenreuther, M. , & Bernatchez, L. (2018). Eco‐evolutionary genomics of chromosomal inversions. Trends in Ecology & Evolution, 33, 427–440. 29731154 10.1016/j.tree.2018.04.002

[eva13587-bib-0102] Wringe, B. F. , Anderson, E. C. , Jeffery, N. W. , Stanley, R. R. , & Bradbury, I. R. (2018). Development and evaluation of SNP panels for the detection of hybridization between wild and escaped Atlantic salmon (*Salmo salar*) in the western Atlantic. Canadian Journal of Fisheries and Aquatic Sciences, 76, 695–704.

[eva13587-bib-0103] Xuereb, A. , Rougemont, Q. , Dallaire, X. , Moore, J. S. , Normandeau, E. , Bougas, B. , Perreault‐Payette, A. , Koop, B. F. , Withler, R. , & Beacham, T. (2022). Re‐evaluating Coho salmon (Oncorhynchus kisutch) conservation units in Canada using genomic data. Evolutionary Applications, 15, 1925–1944. 36426130 10.1111/eva.13489 PMC9679250

