# Supplementary material for: Multifaceted framework for defining conservation units: An example from Atlantic salmon (Salmo salar) in Canada
Source: Evol Appl. 2023 Sep 15;16(9):1568–85. doi: 10.1111/eva.13587 (PMC10519414; doi:10.1111/eva.13587)
Supplement: Supplementary file 1 — Figure S1. [file EVA-16-1568-s001.docx]

Supplementary Materials for

# Multifaceted Framework for defining conservation units: an example from Atlantic salmon (*Salmo salar*) in Canada

**This PDF file includes:**

Supplementary results

Tables S1 to S7

Figs. S1 to S9

Supplemental references

**Supplementary results**

***Evolutionary significance based on climate-linked differences***

Redundancy analysis (RDA; p<0.001) of bioclimatic data in Labrador supports differences between the three genetic clusters (Fig. 6A). Loadings of bioclimatic variables on each axis are provided in Table S4. The first RDA axis primarily separated Southern Labrador from Lake Melville and Northern Labrador, where differences were driven by variation in temperature (specifically minimum temperature, temperature in the coldest quarter, temperature seasonality, and annual temperature range) and precipitation (including precipitation in the coldest quarter, precipitation in driest quarter, and precipitation seasonality). Overall, temperature and precipitation were generally higher and less variable in southern Labrador compared to other regions. The highest loading variable on RDA axis 1 was precipitation of the coldest quarter (bio19) (see Fig. 6B). The second RDA axis separated Lake Melville from northern Labrador, which is primarily driven by the temperature (mean temperature of the driest quarter, maximum temperature, and mean temperature of warmest quarter) and precipitation in the wettest month. Generally, temperature and precipitation were higher in Lake Melville relative to northern Labrador, although temperature during the driest quarter was warmer in northern Labrador than Lake Melville. The highest loading variable on RDA axis 2 was mean temperature of the warmest quarter (bio10) (see Fig. 6C). These results support clear differences in climate that are linked to the three genetic groups which can drive local adaptation.

**Table S1.** Sampling locations for the genomic datasets (220,000 SNP array) for rivers located in Atlantic salmon (Salmo salar) designatable units (DUs). Location coordinates and sample size are provided, as well as sample year and life stage when data were available.

| **DU COSEWIC 2010** | **Site Name** | **Code** | **Lat** | **Long** | **Sample size** | **Sample Year** | **Life stage** |
| --- | --- | --- | --- | --- | --- | --- | --- |
| DU2 | Hunt River | HU | 55.57 | -60.67 | 20 |  |  |
| DU2 | English River | ENG | 54.97 | -59.75 | 28 | 2010 | parr |
| DU2 | Big River | BIG | 54.84 | -58.94 | 28 | 2009 | parr |
| DU2 | Main Brook | MB | 54.24 | -57.87 | 21 | 2013-2014 | parr |
| DU2 | Red Wine River | RW | 53.93 | -61.00 | 22 | 2013-2014 | parr |
| DU2 | Mulligan River | MU | 53.87 | -60.09 | 17 | 2013-2014 | parr |
| DU2 | Crooked River | CR | 53.87 | -60.83 | 21 | 2013-2014 | parr |
| DU2 | Sebaskachu River | SK | 53.79 | -60.14 | 22 | 2013-2014 | parr |
| DU2 | Susan River | SR | 53.74 | -61.04 | 22 | 2013-2014 | parr |
| DU2 | Cape Caribou | CB | 53.62 | -60.42 | 21 | 2013-2014 | parr |
| DU2 | Sand Hill River | SH | 53.57 | -56.35 | 20 |  |  |
| DU2 | Eagle River | EA | 53.53 | -57.47 | 22 |  |  |
| DU2 | Kenamu River | KE | 53.48 | -59.91 | 22 | 2013-2014 | parr |
| DU2 | Paradise River | PA | 53.42 | -57.25 | 20 | 2011 | parr |
| DU2 | Peters River | PR | 53.34 | -60.71 | 21 | 2013-2014 | parr |
| DU2 | Traverspine River | TR | 53.28 | -60.28 | 22 | 2013-2014 | parr |
| DU2 | Caroline River | CL | 53.25 | -60.42 | 20 | 2013-2014 | parr |
| DU2 | St Charles | CHR | 52.23 | -55.84 | 27 | 2011 | parr |
| DU2 | L'anse au Loup River | LL | 51.53 | -56.82 | 22 | 2011 | parr |
| DU2 | Forteau River | FO | 51.48 | -56.94 | 21 | 2011 | parr |
| DU3 | Beaver Brook | BVB | 50.90 | -56.15 | 29 | 2009 | parr |
| DU3 | Great Rattling Brook - Exploits | GRB | 49.62 | -56.17 | 26 | 2010 | parr |
| DU3 | Campbellton | CMP | 49.28 | -54.93 | 25 | 2009 | parr |
| DU3 | Terra Nova River | TNR | 48.67 | -54.00 | 29 | 2009 | parr |
| DU4 | North Brook Trepassey | NBT | 46.74 | -53.36 | 25 | 2010 | parr |
| DU4 | Little Salmonier | LSR | 47.04 | -53.75 | 17 | 2011 | parr |
| DU4 | Northeast Placentia River | NPR | 47.29 | -53.80 | 81 | 2017-2019 | parr |
| DU4 | Ship Harbour Brook | SHI | 47.35 | -53.87 | 84 | 2017-2019 | parr |
| DU4 | Southeast Placentia River | SPR | 47.23 | -53.88 | 97 | 2017-2019 | parr |
| DU4 | Fair Haven Brook | FHB | 47.54 | -53.89 | 103 | 2017-2019 | parr |
| DU4 | Come By Chance River | CBC | 47.97 | -53.96 | 79 | 2017-2019 | parr |
| DU4 | Branch River | BRA | 46.89 | -53.97 | 92 | 2017-2019 | parr |
| DU4 | North Harbour River | NHR | 47.92 | -54.03 | 88 | 2017-2019 | parr |
| DU4 | Little Barasway Brook | LBB | 47.18 | -54.03 | 15 | 2017-2019 | parr |
| DU4 | Great Barasway Brook | GBW | 47.12 | -54.06 | 89 | 2017-2019 | parr |
| DU4 | Lance River | LAN | 46.82 | -54.07 | 9 | 2017-2019 | parr |
| DU4 | Cuslett Brook | CUS | 46.96 | -54.16 | 99 | 2017-2019 | parr |
| DU4 | Black River | BLA | 47.89 | -54.17 | 83 | 2017-2019 | parr |
| DU4 | Pipers Hole River | PHR | 47.93 | -54.27 | 88 | 2017-2019 | parr |
| DU4 | Sandy Harbour River | SHA | 47.71 | -54.36 | 74 | 2017-2019 | parr |
| DU4 | Nonsuch River | NON | 47.45 | -54.64 | 93 | 2017-2019 | parr |
| DU4 | Cape Roger Brook | CRB | 47.44 | -54.69 | 86 | 2017-2019 | parr |
| DU4 | Bay de L'Eau River | BDL | 47.51 | -54.73 | 91 | 2017-2019 | parr |
| DU4 | Rushoon River | RUS | 47.37 | -54.92 | 85 | 2017-2019 | parr |
| DU4 | Long Harbour | LHR | 47.82 | -54.94 | 20 | 2012 | parr |
| DU4 | Red Harbour River East | RHA | 47.33 | -54.99 | 91 | 2017-2019 | parr |
| DU4 | Red Harbour River West | RHW | 47.30 | -55.02 | 78 | 2017-2019 | parr |
| DU4 | Big Salmonier Brook | BSA | 47.06 | -55.22 | 84 | 2017-2019 | parr |
| DU4 | Tides Brook | TDS | 47.13 | -55.26 | 69 | 2017-2019 | parr |
| DU4 | Northwest Brook (MortierBay) | NMB | 47.17 | -55.32 | 87 | 2017-2019 | parr |
| DU4 | Garnish | GAR | 47.23 | -55.35 | 22 | 2009 | parr |
| DU4 | Bay du Nord | BDN | 47.73 | -55.44 | 20 | 2008 | parr |
| DU4 | Lawn River | LWN | 46.95 | -55.54 | 81 | 2017-2019 | parr |
| DU4 | Conne | CNR | 47.91 | -55.70 | 90 | 2010, 2019 | parr |
| DU4 | Taylor Bay Brook (Burin Penn) | TBR | 46.88 | -55.71 | 80 | 2017-2019 | parr |
| DU4 | Piercey's Brook | PBR | 46.88 | -55.86 | 83 | 2017-2019 | parr |
| DU4 | Dollards Brook | DLR | 48.02 | -56.57 | 26 | 2016 | parr |
| DU4 | Isle aux Morts River | IAM | 47.59 | -59.01 | 28 | 2011 | parr |
| DU5 | Humber River | TYB | 49.55 | -57.10 | 29 | 2009 | parr |
| DU5 | Flat Bay Brook | FLB | 48.41 | -58.58 | 24 | 2009 | parr |
| DU5 | Little Codroy River | COD | 47.77 | -59.27 | 28 | 2009 | parr |
| DU6 | Western Arm | WAB | 51.19 | -56.76 | 18 | 2016 | adults |
| DU6 | Big East | BER | 50.63 | -57.17 | 27 | 2009 | parr |
| DU6 | Trout River | TRE, TRF, TRN, TRW | 49.64 | -57.75 | 27 | 2019 | parr |
| DU8 | Corneille | COR | 50.28 | -62.88 | 28 | 2018 | adult |
| DU8 | Saint-Jean (NorthShore) SJQ8 | SJQ | 50.28 | -64.33 | 28 | 2018 | adult |
| DU8 | Natashquan | NAT | 50.12 | -61.80 | 28 | 2018 | adult |
| DU8 | Riviere Aux Rochers | ARO | 50.00 | -66.86 | 48 | 2012 | adult |
| DU8 | Riviere de la Trinite | TRI | 49.42 | -67.30 | 49 | 2012 | adult |
| DU9 | Jupiter | JUP | 49.47 | -63.58 | 28 | 2018 | adult |
| DU10 | A mars | aMars | 48.34 | -70.88 | 26 | 2018 | adult |
| DU12 | Madeleine | MAD | 49.23 | -65.32 | 28 | 2018 | adult |
| DU12 | Matapedia | MAT | 48.18 | -67.14 | 15 | 2018 | parr |
| DU12 | Kedgwick | KED | 47.91 | -67.91 | 15 | 2018 | parr |
| DU12 | Patapedia | PAT | 47.86 | -67.39 | 24 | 2018 | parr |
| DU12 | Upsalquitch | UPS | 47.57 | -66.54 | 28 | 2018 | parr |
| DU12 | Miramichi-Upper Northwest | MUN | 47.17 | -65.94 | 24 | 2016 | parr |
| DU12 | Kouchibouguac | KOU | 46.74 | -65.20 | 31 | 2018 | parr |
| DU12 | CheticampRiver | CHT | 46.64 | -60.95 | 12 | 2018 | parr |
| DU12 | Northwest Complex(PEI) | NWP | 46.63 | -64.04 | 17 | 2018 | parr |
| DU12 | Miramichi-Upper Southwest | MSW | 46.55 | -66.04 | 23 | 2016 | parr |
| DU12 | Northeast Margaree | MNE | 46.47 | -60.92 | 12 | 2018 | parr |
| DU12 | Northeast Complex-1 (PEI) | NEP | 46.45 | -62.21 | 27 | 2018 | parr |
| DU12 | Northeast Complex-2 (PEI) | NET | 46.38 | -62.57 | 24 | 2018 | parr |
| DU12 | Richibucto | RIC | 46.36 | -65.15 | 31 | 2018 | parr |
| DU12 | Morells | MOR | 46.30 | -62.71 | 18 | 2018 | parr |
| DU12 | South Central PEI | SCP | 46.28 | -63.49 | 14 | 2018 | parr |
| DU12 | Southwest Margaree | MRS | 46.24 | -61.12 | 14 | 2018 | parr |
| DU12 | Mabou River | MAB | 46.04 | -61.31 | 27 | 2018 | parr |
| DU12 | Graham River | JGC | 45.86 | -61.49 | 11 | 2018 | parr |
| DU12 | River Philip | RPH | 45.59 | -63.82 | 17 | 2018 | parr |
| DU12 | East River Pictou | PIE | 45.54 | -62.88 | 23 | 2018 | parr |
| DU13 | Clyburn | CLY | 46.66 | -60.41 | 28 | 2019 |  |
| DU13 | Baddeck | BAD | 46.10 | -60.84 | 28 | 2016 | parr |
| DU13 | Inhabitants River | INH | 45.60 | -61.23 | 28 | 2016 | parr |
| DU14 | Sheet Harbour West River | WES | 44.95 | -62.59 | 28 | 2019 | smolt |
| DU14 | LaHave | LAH | 44.37 | -64.50 | 22 |  |  |
| DU15 | Big Salmon | BSR | 45.42 | -65.41 | 22 | 2014 |  |
| DU15 | North River NS | NRH | 45.38 | -63.31 | 22 |  |  |
| DU15 | Stewiacke | STW | 45.14 | -63.38 | 22 | 2014 |  |
| DU15 | Gaspereau River | GAK | 45.06 | -64.38 | 26 | 2016 |  |
| DU16 | Nashwaak | NSH | 45.96 | -66.62 | 20 | 2006-2009 |  |

**Table S2.** Sampling locations for 101-microsatellite dataset for Atlantic salmon (Salmo salar) located in Labrador, Canada (for details see Bradbury et al., 2018). Location codes, sample size, and sampling year are provided.

| **Location** | **Code** | **Sample** | **Year** |
| --- | --- | --- | --- |
| Hunt River | HUN | 49 | 2014 |
| English River | ENG | 60 | 2010 |
| Big River | BIG | 50 | 2009 |
| Pottle's Bay | POT | 13 | 2016 |
| West Brook | WST | 31 | 2016 |
| Middle Brook | MDB | 50 | 2016 |
| Tom Luscombe | TLU | 50 | 2016 |
| Partridge Point | PPB | 50 | 2016 |
| Double Mer | DBMLR | 50 | 2016 |
| Mulligan River | MUL | 50 | 2014 |
| Sebaskatchu River | SEB | 30 | 2014 |
| Crooked River | CRO | 51 | 2014 |
| Red Wine River | RWR | 50 | 2014 |
| Susan River | SUS | 50 | 2014 |
| Cape Caribou | CCRM | 42 | 2014 |
| Caroline River | CAR | 25 | 2014 |
| Traverspine River | TSP | 50 | 2014 |
| Kenamich River | KEN | 30 | 2016 |
| Kenamu River | KMU | 18 | 2014 |
| Main Brook | MNB | 42 | 2014 |
| Eagle River | EGR | 50 | 2011 |
| Paradise River | PARR | 40 | 2011 |
| Paradise Brook | PARA | 42 | 2011 |
| Muddy Bay Brook | MBB | 50 | 2011 |
| Sand Hill | SAN | 50 | 2010 |
| Alexis River | ALR | 50 | 2009 |
| Shinny's River | SHIN | 50 | 2011 |
| St. Lewis River | STL | 50 | 2011 |
| Port Marnum | PTM | 33 | 2011 |
| Mary's Harbour | MH | 50 | 2011 |
| Charles River | CHA | 50 | 2011 |
| Pinware River | PIN | 50 | 2010 |
| L'anse au Loop | LL | 50 | 2011 |
| Forteau River | FORT | 50 | 2011 |

**Table S3.** Sampling locations for 96-SNP dataset for Atlantic salmon (Salmo salar) located in Labrador, Canada. Location coordinates, data source, and sample size are provided, as well as sample year and life stage when data were available.

| **River** | **Pop Code** | **Lat** | **Long** | **Sample Size** | **Data Source** | **Year** | **Life stage** |
| --- | --- | --- | --- | --- | --- | --- | --- |
| Webb Brook | WBB | 56.80 | -61.91 | 31 | Jeffery et al. 2018 | 2011 | parr |
| Hunt River | HU | 55.57 | -60.67 | 20 | Moore et al. 2014 | 2009 | parr & smolt |
| River 72 | R72 | 55.12 | -60.10 | 50 | unpublished | 2017 | parr |
| Makkovik Brook | MKB | 55.05 | -59.16 | 47 | unpublished | 2017 | parr |
| English River | ENG | 54.97 | -59.75 | 33 | Jeffery et al. 2018 | 2010 | parr |
| Makkovik River | MKR | 54.96 | -59.43 | 50 | unpublished | 2017 | parr |
| Adlavik Brook | ADL | 54.84 | -59.14 | 49 | unpublished | 2017 | parr |
| Big River | BIG | 54.84 | -58.94 | 26 | Jeffery et al. 2018 | 2009 | parr |
| Rattling Brook | RAT | 54.78 | -58.95 | 50 | unpublished | 2017 | parr |
| Pamiulik River | PAM | 54.72 | -58.58 | 46 | unpublished | 2017 | parr |
| Jeanette Bay Brook | JBB | 54.72 | -58.09 | 42 | unpublished | 2017 | parr |
| South Brook | SBR | 54.71 | -59.91 | 47 | unpublished | 2017 | parr |
| Michael River | MIC | 54.67 | -57.84 | 50 | unpublished | 2017 | parr |
| Pottle's Bay | PBB | 54.48 | -57.73 | 21 | unpublished | 2016 | parr |
| West Brook | WBL | 54.40 | -58.10 | 20 | unpublished | 2016 | parr |
| Tom Luscombe | TOM | 54.34 | -58.55 | 20 | unpublished | 2016 | parr |
| Main Brook | MB | 54.24 | -57.87 | 21 | Sylvester et al. 2018 | 2013 or 2014 | parr |
| Partridge Point | PPB | 54.10 | -59.48 | 21 | unpublished | 2016 | parr |
| Double Mer | DBL | 54.02 | -59.65 | 21 | unpublished | 2016 | parr |
| Red Wine River | RW | 53.93 | -61.00 | 22 | Sylvester et al. 2018 | 2013 or 2014 | parr |
| Mulligan River | MU | 53.87 | -60.09 | 21 | Sylvester et al. 2018 | 2013 or 2014 | parr |
| Crooked River | CR | 53.87 | -60.83 | 21 | Sylvester et al. 2018 | 2013 or 2014 | parr |
| Sebaskachu River | SK | 53.79 | -60.14 | 22 | Sylvester et al. 2018 | 2013 or 2014 | parr |
| Susan River | SR | 53.74 | -61.04 | 22 | Sylvester et al. 2018 | 2013 or 2014 | parr |
| Muddy Bay Brook | MBB | 53.64 | -57.07 | 34 | Jeffery et al. 2018 | 2011 | parr |
| Cape Caribou | CB | 53.62 | -60.42 | 21 | Sylvester et al. 2018 | 2013 or 2014 | parr |
| Sand Hill River | SH | 53.57 | -56.35 | 20 | Sylvester et al. 2018 |  |  |
| Eagle River | EA | 53.53 | -57.47 | 22 | Sylvester et al. 2018 | 2011 | parr |
| Kenamu River | KE | 53.48 | -59.91 | 22 | Sylvester et al. 2018 | 2013 or 2014 | parr |
| Paradise River | PA | 53.42 | -57.25 | 20 | Sylvester et al. 2018 | 2011 | parr |
| Southwest Brook | SW | 53.42 | -57.23 | 25 | Moore et al. 2014 | 2004 | adults |
| Peters River | PR | 53.34 | -60.71 | 21 | Sylvester et al. 2018 | 2013 or 2014 | parr |
| Kenemich River | KNM | 53.32 | -59.82 | 20 | unpublished | 2016 | parr |
| Traverspine River | TR | 53.28 | -60.28 | 22 | Sylvester et al. 2018 | 2013 or 2014 | parr |
| Caroline River | CL | 53.25 | -60.42 | 20 | Sylvester et al. 2018 | 2013 or 2014 | parr |
| Hawke River | HWK | 53.03 | -56.06 | 31 | Jeffery et al. 2018 | 2011 | parr |
| Alexis | ALX | 52.60 | -56.53 | 34 | Jeffery et al. 2018 | 2009 | parr |
| Shinnys | SHR | 52.59 | -56.34 | 34 | Jeffery et al. 2018 | 2011 | parr |
| St. Lewis | SLW | 52.43 | -56.17 | 34 | Jeffery et al. 2018 | 2011 | parr |
| Port Marum | PMR | 52.40 | -55.74 | 33 | Jeffery et al. 2018 | 2011 | parr |
| St Charles | CHR | 52.23 | -55.84 | 34 | Jeffery et al. 2018 | 2011 | parr |
| Pinware | PIN | 51.63 | -56.69 | 34 | Jeffery et al. 2018 | 2010 | parr |
| L'anse au Loup River | LL | 51.53 | -56.82 | 22 | Sylvester et al. 2018 | 2011 | parr |
| Forteau River | FOR | 51.48 | -56.94 | 34 | Moore et al. 2014 | 2011 | parr |
| St Paul River | STP | 51.47 | -57.70 | 25 | Bourret et al. 2013 | 2004 |  |
| Vieux Fort | VF | 51.32 | -58.02 | 25 | Moore et al. 2014 | 2004 | adults |
| Napetipi | NAP | 51.30 | -58.05 | 25 | Moore et al. 2014 | 2004 | adults |

**Table S4.** Bioclimatic variables downloaded from WorldClim (Fick & Hijmans, 2017) using the R package rbioclim (Exposito-Alonso, 2017). The bioclimatic variables were standardized to a mean of 0 and a standard deviation of 1 for analyses. Loadings of bioclimatic variables on each axis of the redundancy analysis (RDA) are provided.

| **Name** | **RDA1** | **RDA2** | **Description** |
| --- | --- | --- | --- |
| bio1 | 1.030 | -0.568 | Annual mean temperature |
| bio2 | -1.168 | -0.664 | Mean diurnal temperature range - Mean of the monthly (maximum temperature - minimum temperature) |
| bio3 | 0.882 | -0.672 | Isothermality - Mean diurnal temperature range (bio2) / Annual temperature range (bio7) |
| bio4 | -1.332 | -0.302 | Temperature seasonality - Standard deviation of the annual mean temperature |
| bio5 | -0.981 | -0.879 | Maximum temperature - Maximum temperature of the warmest month |
| bio6 | 1.311 | 0.040 | Minimum temperature - Minimum temperature of the coldest month |
| bio7 | -1.304 | -0.397 | Annual temperature range - Maximum temperature (bio5) - minimum temperature (bio6) |
| bio8 | -1.160 | -0.004 | Mean temperature of wettest quarter |
| bio9 | 0.709 | 0.942 | Mean temperature of driest quarter |
| bio10 | -0.498 | -1.042 | Mean temperature of warmest quarter |
| bio11 | 1.307 | -0.046 | Mean temperature of coldest quarter |
| bio12 | 1.195 | -0.514 | Annual precipitation |
| bio13 | 0.142 | -0.764 | Precipitation of wettest month |
| bio14 | 1.144 | -0.687 | Precipitation of driest month |
| bio15 | -1.306 | 0.207 | Precipitation seasonality - Coefficient of variation of the monthly precipitation |
| bio16 | 0.121 | -0.563 | Precipitation of wettest quarter |
| bio17 | 1.322 | -0.444 | Precipitation of driest quarter |
| bio18 | -0.064 | -0.500 | Precipitation of warmest quarter |
| bio19 | 1.334 | -0.122 | Precipitation of coldest quarter |

**Table S5.** Results from STRUCTURE HARVESTER for STRUCTURE runs of the 101-microsatellite and 96-SNPs datasets. Best K based on Evanno’s delta K (Evanno et al., 2005) is highlighted in gray for each dataset.

| **101-microsatellites** | | | | | | |
| --- | --- | --- | --- | --- | --- | --- |
| **K** | **Reps** | **Mean LnP(K)** | **Stdev LnP(K)** | **Ln'(K)** | **\|Ln''(K)\|** | **Delta K** |
| 1 | 3 | -287758.10 | 0.44 | NA | NA | NA |
| 2 | 3 | -283577.10 | 2.52 | 4181.00 | 1869.77 | 740.83 |
| 3 | 3 | -281265.87 | 5.85 | 2311.23 | 568.97 | 97.22 |
| 4 | 3 | -279523.60 | 164.52 | 1742.27 | 488.93 | 2.97 |
| 5 | 3 | -277292.40 | 7.80 | 2231.20 | 1293.97 | 165.96 |
| 6 | 3 | -276355.17 | 616.47 | 937.23 | 342.60 | 0.56 |
| 7 | 3 | -275075.33 | 8.10 | 1279.83 | 586.53 | 72.43 |
| 8 | 3 | -274382.03 | 16.41 | 693.30 | 6.40 | 0.39 |
| 9 | 3 | -273695.13 | 280.19 | 686.90 | 276.43 | 0.99 |
| 10 | 3 | -273284.67 | 75.21 | 410.47 | NA | NA |
| **96-SNPs** | | | | | | |
| **K** | **Reps** | **Mean LnP(K)** | **Stdev LnP(K)** | **Ln'(K)** | **\|Ln''(K)\|** | **Delta K** |
| 1 | 3 | -89760.87 | 0.06 | NA | NA | NA |
| 2 | 3 | -88634.00 | 1.90 | 1126.87 | 380.37 | 200.19 |
| 3 | 3 | -87887.50 | 11.20 | 746.50 | 649.70 | 58.01 |
| 4 | 3 | -87790.70 | 46.54 | 96.80 | 197.87 | 4.25 |
| 5 | 3 | -87496.03 | 28.83 | 294.67 | 52.27 | 1.81 |
| 6 | 3 | -87253.63 | 80.33 | 242.40 | 114.73 | 1.43 |
| 7 | 3 | -87125.97 | 51.72 | 127.67 | 186.47 | 3.60 |
| 8 | 3 | -86811.83 | 28.16 | 314.13 | 148.53 | 5.28 |
| 9 | 3 | -86646.23 | 22.89 | 165.60 | 1.60 | 0.07 |
| 10 | 3 | -86482.23 | 26.08 | 164.00 | NA | NA |

**Table S6.** Frequency of karyotypes associated with a chromosomal rearrangement (Ssa01/Ssa23) within Atlantic salmon populations in Labrador (Lake Melville and coastal Labrador). Proportion are derived from Lehnert et al. (2019).

| **Pop** | **Translocation (North American standard)** | **Heterozygote** | **No translocation**  **(European standard)** | **Frequency of EU chromosome** |
| --- | --- | --- | --- | --- |
| **Lake Melville** | |  |  |  |
| CB | 0.52 | 0.19 | 0.29 | 0.38 |
| CL | 0.55 | 0.30 | 0.15 | 0.30 |
| CR | 0.67 | 0.24 | 0.10 | 0.21 |
| KE | 0.32 | 0.18 | 0.50 | 0.59 |
| MB | 0.76 | 0.19 | 0.05 | 0.14 |
| MU | 0.82 | 0.18 | 0.00 | 0.09 |
| RW | 0.55 | 0.18 | 0.27 | 0.36 |
| SK | 0.55 | 0.41 | 0.05 | 0.25 |
| SR | 0.41 | 0.32 | 0.27 | 0.43 |
| TR | 0.45 | 0.23 | 0.32 | 0.43 |
|  |  |  | **Mean** | **0.32** |
| **Coastal Labrador** | |  |  |  |
| PA | 0.90 | 0.05 | 0.05 | 0.08 |
| LL | 0.73 | 0.14 | 0.14 | 0.20 |
| EA | 0.86 | 0.05 | 0.09 | 0.11 |
| FO | 0.90 | 0.10 | 0.00 | 0.05 |
| HU | 0.60 | 0.20 | 0.20 | 0.30 |
| SH | 0.75 | 0.20 | 0.05 | 0.15 |
|  |  |  | **Mean** | **0.15** |

**Table S7.** Summary of revised DUs for anadromous Atlantic salmon in Labrador. Support for the discreteness and evolutionary significance criteria for defining DUs are summarized.

| **Proposed DU** | **Support for DU** | |
| --- | --- | --- |
|  | **Discreteness** | **Significance** |
| **DU 02-A Northern Labrador** | - Microsatellites (15, 101) separate coastal Labrador (north DU-02A and south DU-02C) from Lake Melville (DU-02B) at K=2 - 96-SNP dataset separates this DU as one of 3 discrete areas in Labrador - Limited gene flow with adjacent northern DU-01 (Nunavik) based on microsatellites and SNP studies (Moore et al., 2014; Bradbury et al., 2021) - Natural geographic separation (~650 km of coastline) from northern DU-01 (Nunavik) | **Genomic evidence of adaptation (Lehnert et al. 2021):**   - Genomic data separates coastal Labrador from Lake Melville - Genomic differences associated with fatty acid homeostasis distinguish Lake Melville (DU-02B) from coastal areas of Labrador based on gene ontology analyses - Genetic-environment associations delineating coastal Labrador from Lake Melville - Lower frequency of European type Ssa01/Ssa23 chromosomal rearrangement in coastal Labrador compared to Lake Melville (DU 02B) - Genomic differences between populations in Labrador and Nunavik DU-01 have been reported using ~3000 genome-wide SNPs (Bourret et al. 2013)   **Life history:**   - Later run timing compared to other regions of Labrador - Lower incidence of maturation after 1SW compared to Lake Melville and southern Labrador DUs - Older smolt age compared to Lake Melville DU - This DU also differs from the Nunavik DU-01 to the north, which is characterized by slower freshwater growth and older ages at smoltification than other DUs. Salmon within the Nunavik DU also show local migratory routes and life history phenotypes (high seas and estuarine migrants) distinct to these populations   **Climate-linked differences:**   - Differences in temperature (colder) and precipitation (lower) other Labrador DUs - The Northern Labrador DU also differs from the Nunavik DU, which is at the northern extreme of the species’ range in Canada, Arctic-like conditions, rivers discharge south to north, differing from other regions in Canada.   **Additional considerations:**   - Different riverine fish communities (dominated by Arctic Charr) compared to other Labrador DUs - Highest river gradients of rivers in Labrador - Geographic separation (~650 km of coastline) from northern DU-01 (Nunavik) suggesting independent evolutionary histories. |
| **DU 02B Lake Melville** | - Microsatellites (15, 101) separate coastal Labrador (north and south DUs) from this DU at K=2 - 96-SNP dataset separates this DU (Lake Melville) as one of 3 discrete areas in Labrador | **Genomic evidence of adaptation (Lehnert et al. 2021):**   - Genomic data separates Lake Melville from coastal Labrador - Genomic differences associated with fatty acid homeostasis distinguish Lake Melville (DU-02B) from coastal areas of Labrador based on gene ontology analyses - Genetic-environment associations delineating Lake Melville from coastal Labrador - Higher frequency of European type Ssa01/Ssa23 chromosomal rearrangement in Lake Melville compared to coastal Labrador   **Life history:**   - Earlier run timing in this DU compared to coastal regions of Labrador - Incidence of maturation after 1SW intermediate to southern and northern Labrador - Differences in migration routes, access via Lake Melville inlet. - Younger smolts compared to coastal Labrador   **Climate-linked differences:**   - Cooler and more variable temperatures as well as lower and more variable precipitation in Lake Melville (and north Labrador) compared to DU-02C - Generally warmer temperature and higher precipitation in Lake Melville compared to DU-02A, except during the driest quarter where temperatures are cooler in Lake Melville.   **Additional considerations:**   - Differences in riverine fish communities, Lake Melville rivers generally have Atlantic Salmon and sea-run Brook Trout - Lowest river gradients of Labrador region - Lake Melville is a fjord-type estuary, extending >150 km inland from the coast, has a shallow sill at its outlet to Groswater Bay (Labrador Sea) |
| **DU 02C Southern Labrador** | - Microsatellites (15, 101) separate coastal Labrador (north and south DUs) from Lake Melville at K=2 - 96-SNP dataset separates this DU (southern Labrador) as one of 3 discrete areas in Labrador - Limited gene flow with DU to the south (DU-07) based on microsatellites and SNPs studies (Dionne et al., 2008; Moore et al., 2014; Bradbury et al., 2021) | **Genomic evidence of adaptation:**   - Genomic data separates coastal Labrador from Lake Melville - Genomic differences associated with fatty acid homeostasis distinguish Lake Melville (DU-02B) from coastal areas of Labrador based on gene ontology analyses - Genetic-environment associations delineating coastal Labrador from Lake Melville - Lower frequency of European type Ssa01/Ssa23 chromosomal rearrangement in coastal Labrador compared to Lake Melville (DU-02B) - Genomic differences between populations in southern Labrador and neighbouring DU in Quebec (DU-07) have been reported using ~3000 genome-wide SNPs (Bourret et al. 2013)     **Life history:**   - Run timing is intermediate compared to other regions of Labrador - Lower incidence of maturation after 1SW compared to Lake Melville - Differences in migration routes - Potentially younger sea age and smaller size at maturity than northern Labrador - Older smolts compared to Lake Melville - Differences in migration route from other Labrador regions, likely reflecting distance to feeding grounds - Populations in this DU are generally characterized by a longer generation time (6 years) compared to nearby populations in Quebec Eastern North Shore DU-07 (5 years) due to differences in age of smoltification   **Climate-linked differences:**   - Differences in temperature (warmer and less variable) and precipitation (higher and less variable) from other regions in Labrador - This DU is also characterized by colder temperatures than the neighbouring Quebec Eastern North Shore DU-07   **Additional considerations:**   - Differences in fish communities: Brook Trout, Arctic Charr, and Atlantic Salmon are represented more equally in southern Labrador than other regions of Labrador - Intermediate river gradients in this region compared to other parts of Labrador |

**Supplemental Figures**


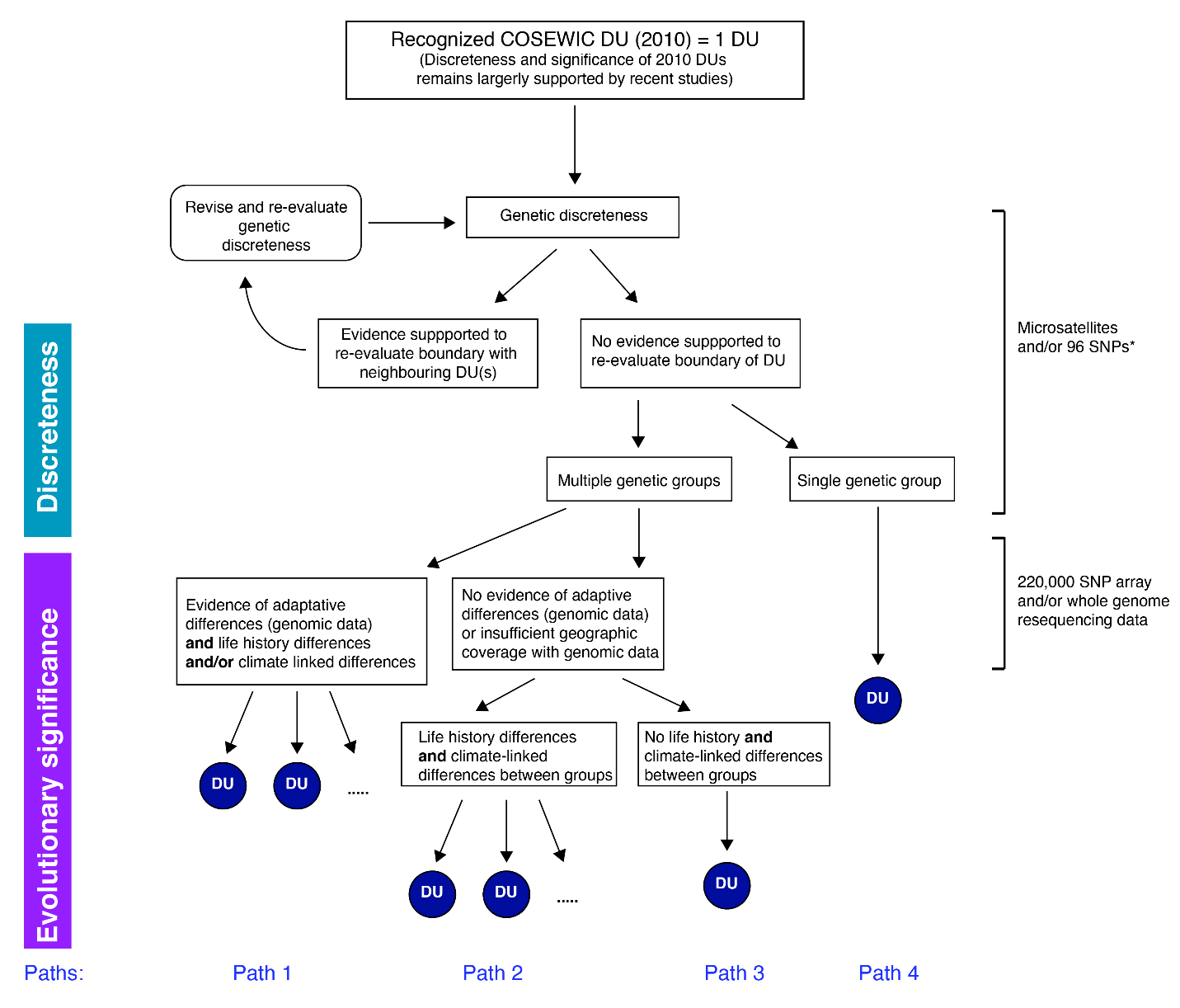


**Figure S1**. Original decision tree used to evaluate discreteness and evolutionary significance of Atlantic salmon DUs (Lehnert et al., 2023).


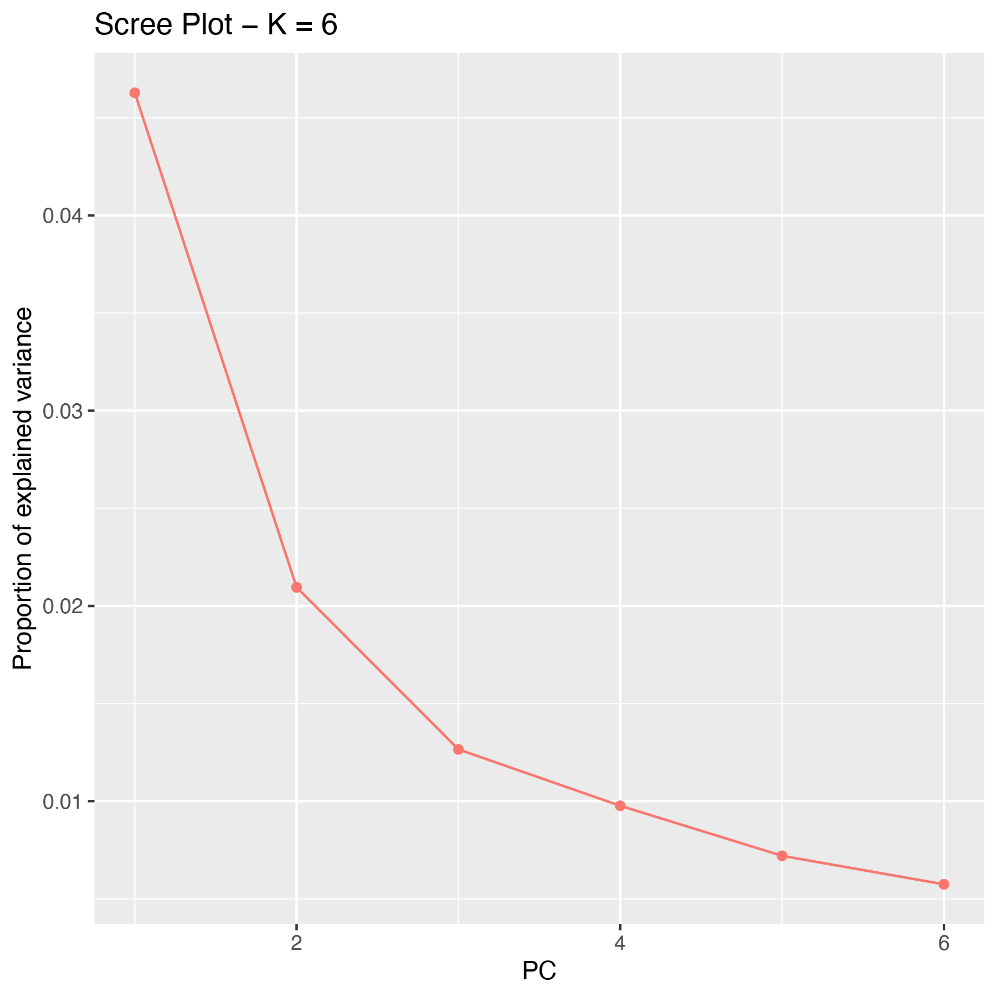


**Figure S2**. Scree plot showing proportion of variance explained for principal component axes (PCs) 1 to 6 based on genomic dataset (220,000 SNP array) for Atlantic salmon (*Salmo salar*) used in the R package *pcadapt* (Luu et al., 2017).


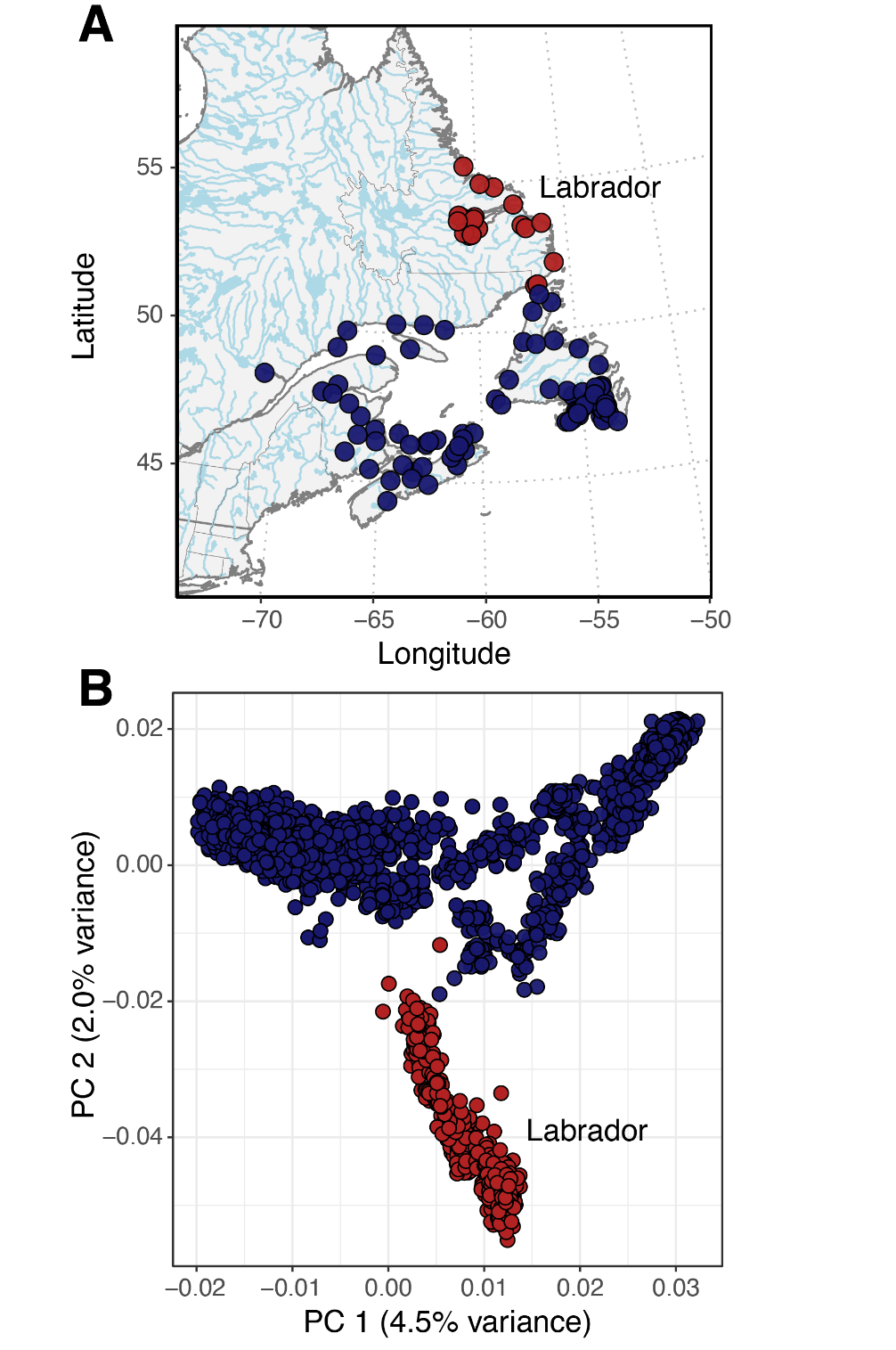


**Figure S3.** **(A)** Map of Atlantic salmon (*Salmo salar*) sampling locations for the genomic dataset (220,000 SNP array) used for unsupervised analyses. **(B)** Broad-scale genetic structure of Atlantic salmon populations based principal component analysis (PCA) of neutral genomic dataset. Populations in Labrador are highlighted by red. Features in maps were generated using data from [https://www.naturalearthdata.com/](https://www.naturalearthdata.com/downloads/) and plotted using the R package *ggplot*.

***
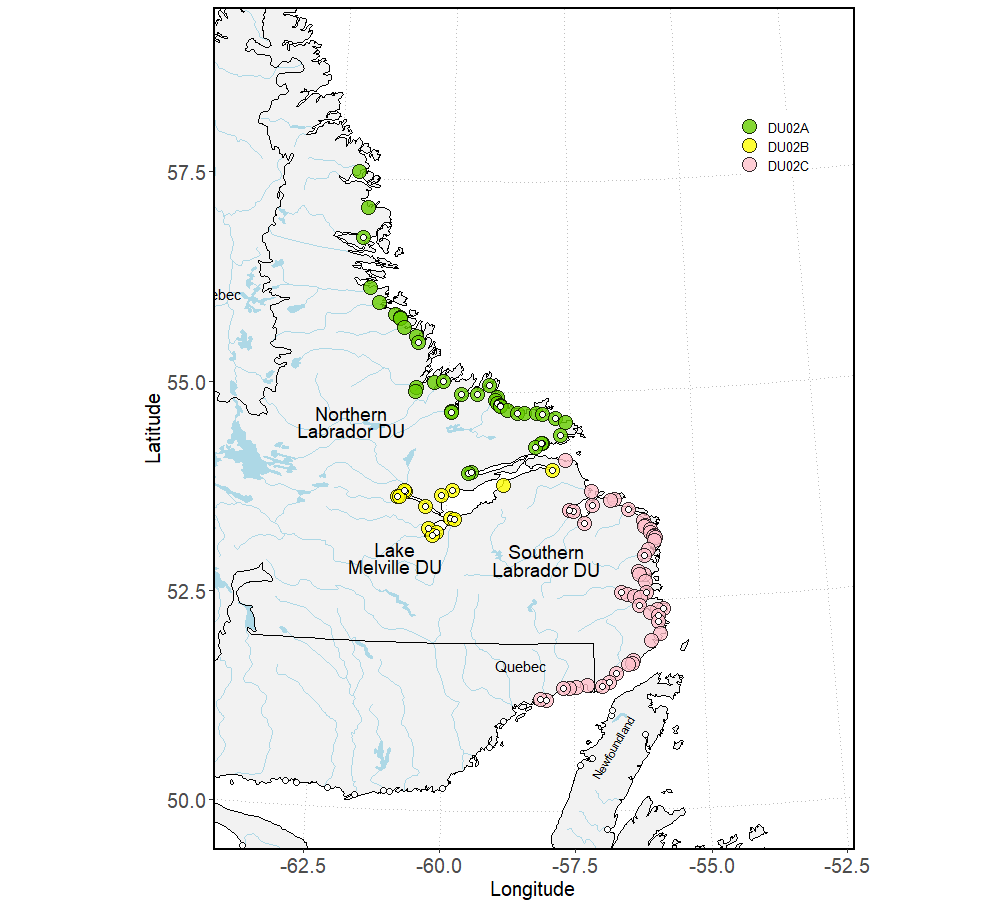
***

**Figure S4.** Map of Atlantic salmon-bearing rivers in Labrador with colours representing the three discrete genetic groups identified in our analyses. Features in maps were generated using data from https://www.naturalearthdata.com/ and plotted using the R package ggplot.


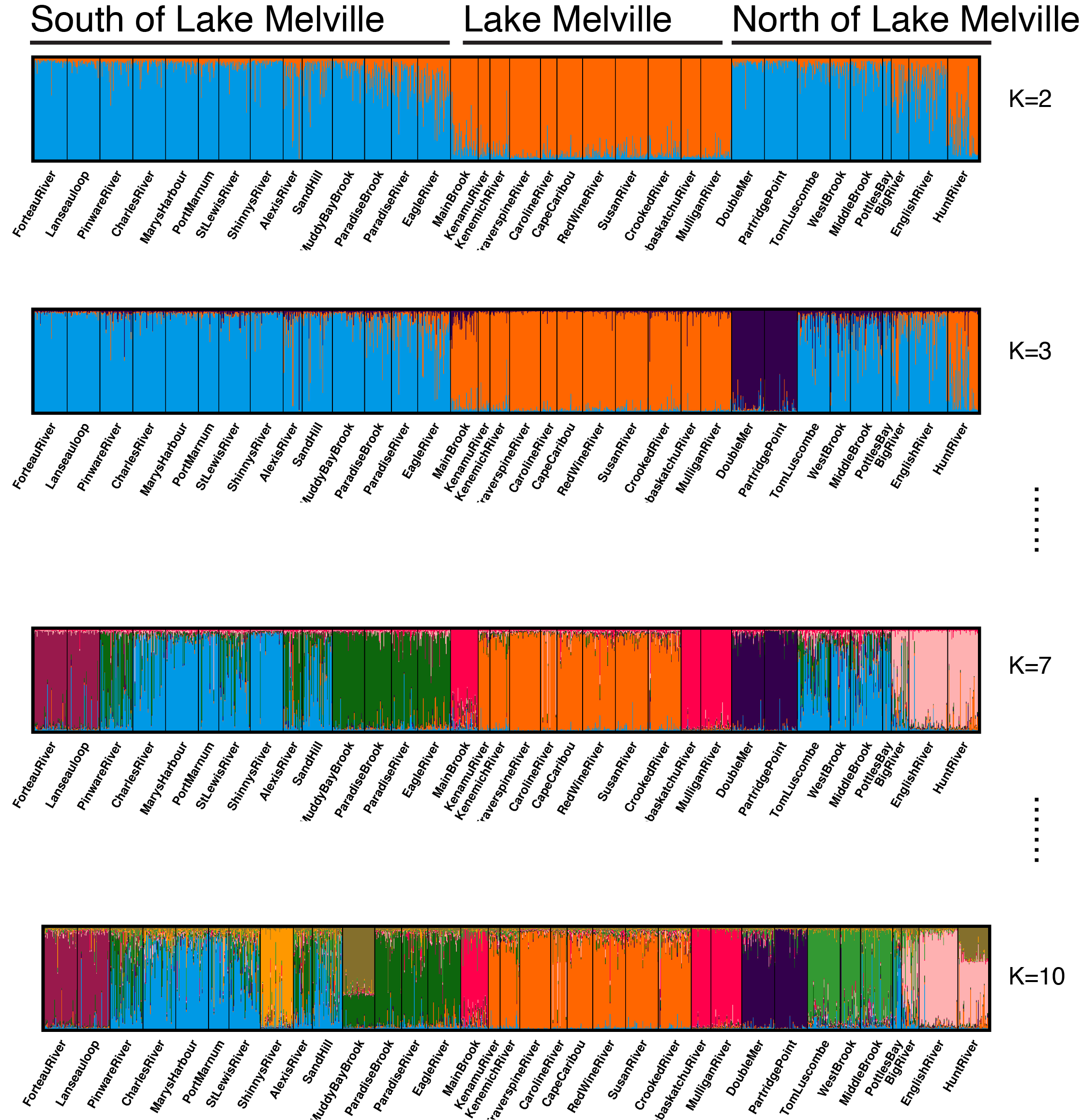


**Figure S5**. Results from STRUCTURE for Labrador using the 101 microsatellites showing genetic clusters K=2 to K=10. We tested values of K ranging from 1 to 10. Best K in STRUCTURE was 2, but K values beyond K=2 were supported and additional structuring was observed beyond K=10. At K=2, Lake Melville sites were clearly separated from other sites in Labrador. At higher values of K, various rivers or geographic regions formed their own clusters.


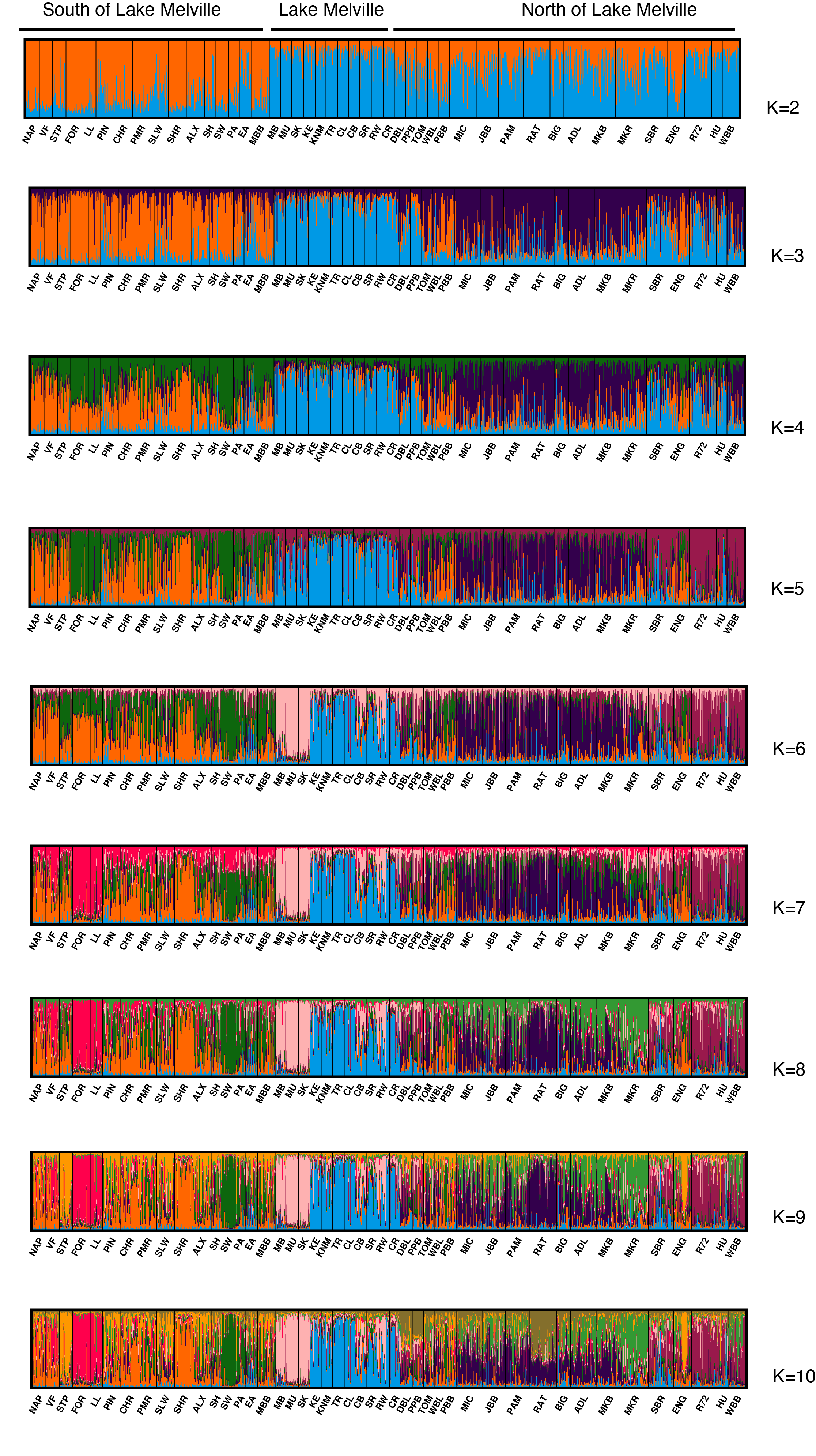


**Figure S6.** Results from STRUCTURE using the 96-SNP dataset with genetic clusters K=2 to K=10. We tested values of K ranging from 1 to 10. Best K in STRUCTURE was 2, but K values beyond K=2 were supported and additional structuring was observed. Sites south of Lake Melville generally clustered separately from sites from Lake Melville and those northward at K=2. At K=3, the DU was separated into three clusters (south Labrador, Lake Melville, and north Labrador). Further clustering of individual rivers and geographic region was apparent at higher values of K.


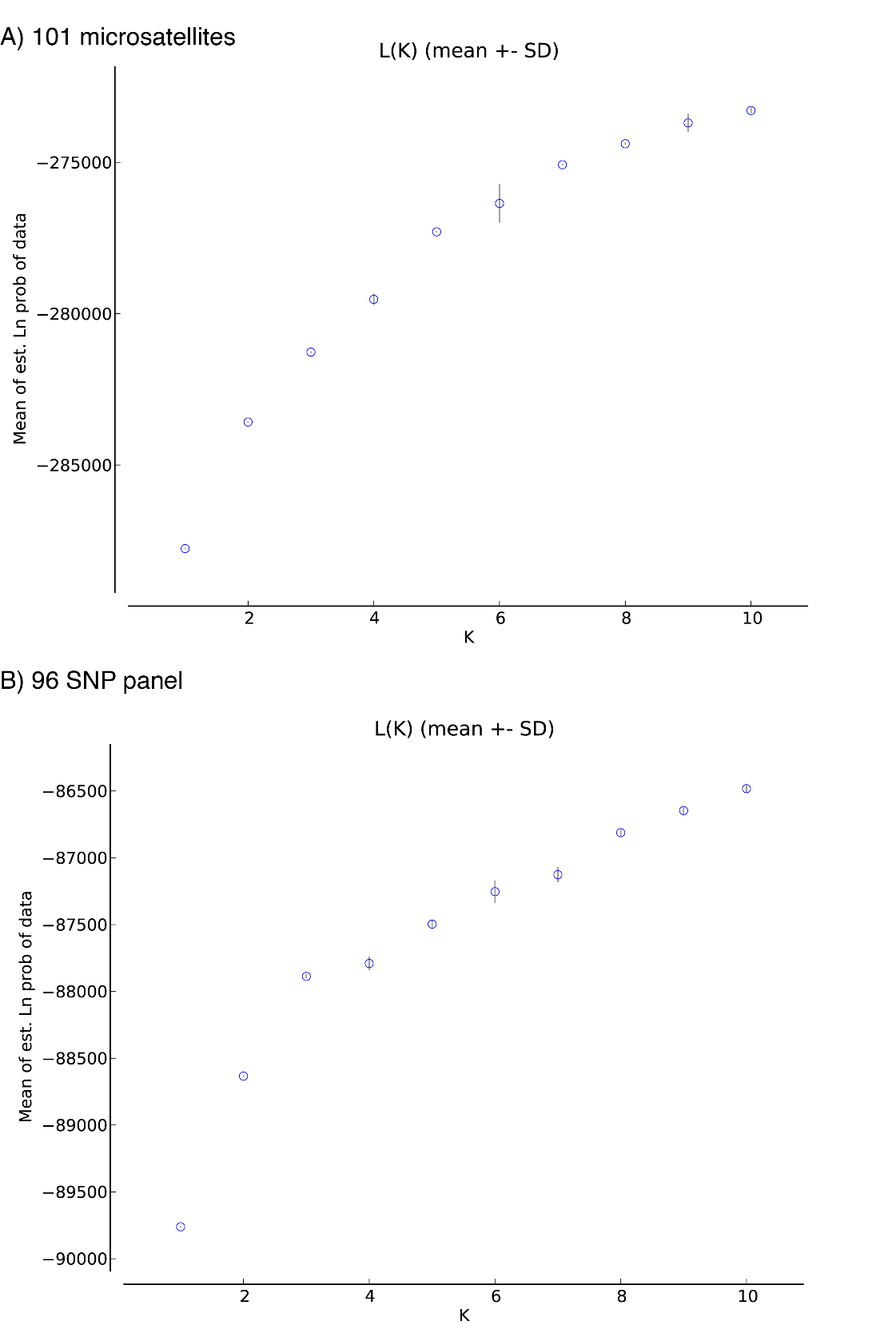


**Figure S7.** Plots generated from STRUCTURE HARVESTER for **(A)** 101-microsatellite and **(B)** 96-SNP datasets. Plots were used to examined the plateau in mean LnPr(*X*|*K*) estimates, which can be used to assess support for the number of genetic clusters present in the dataset.

**Figure S8.** Results of gene ontology analysis based on biological processes that were significantly overrepresented in the outlier data for Labrador. These processes were associated with genes located within 10,000 bp of outlier SNPs (314 SNPs based on K=2 in pcadapt). Outliers are those that differentiate Lake Melville sites from other sites in coastal Labrador. Higher level processes overrepresented in the analysis are indicated by different colour squares in the REVIGO treemap.


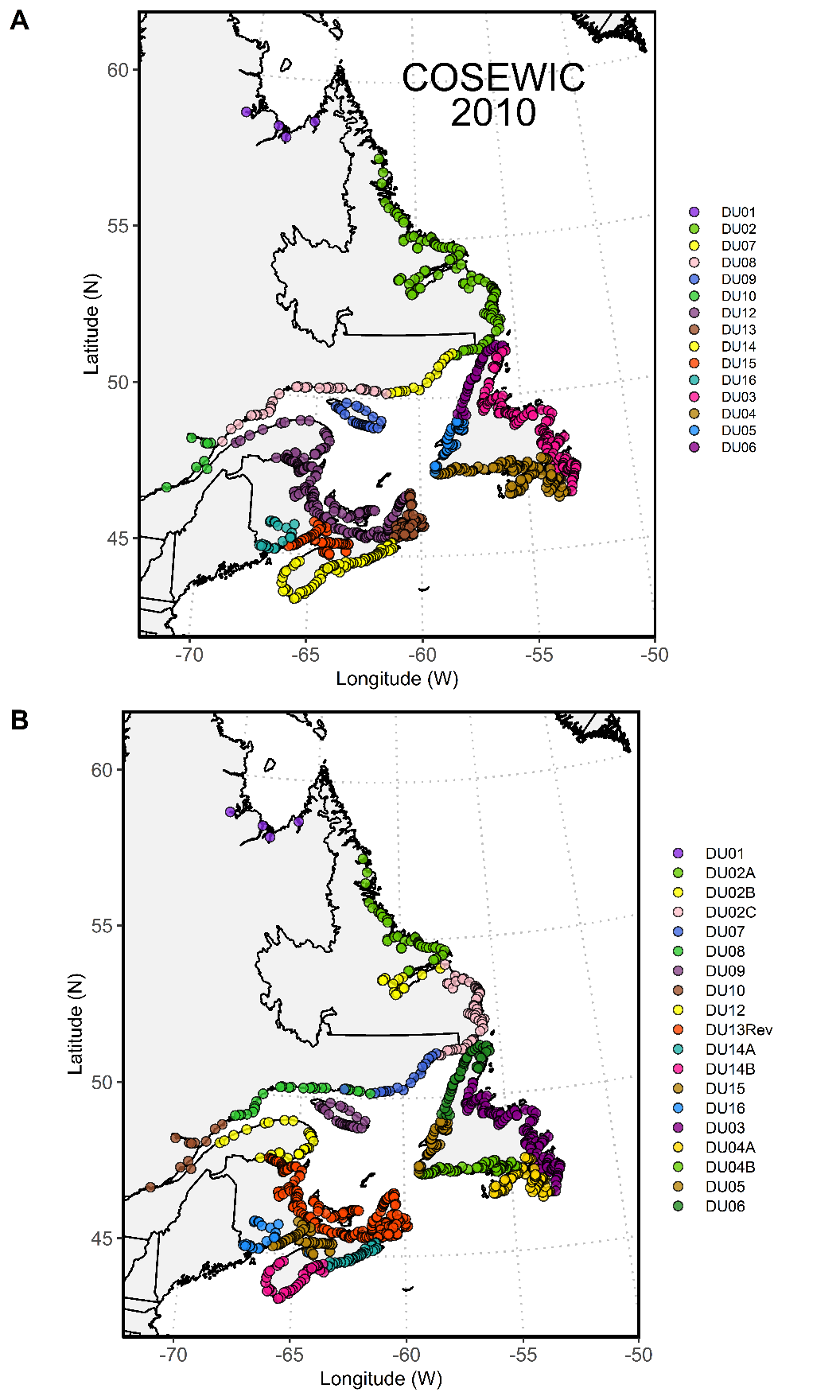


**Figure S9**. Map showing the (A) 2010 COSEWIC DUs and (B) the recent updated COSEWIC DUs for Atlantic salmon.

**Supplemental references**

Bradbury, I. R., Wringe, B. F., Watson, B., Paterson, I., Horne, J., Beiko, R., Lehnert, S. J., Clément, M., Anderson, E. C., Jeffery, N. W., Duffy, S., Sylvester, E. R., Martha, & Bentzen, P. (2018). Genotyping‐by‐sequencing of genome‐wide microsatellite loci reveals fine‐scale harvest composition in a coastal Atlantic salmon fishery. *Evolutionary Applications*, **11**, 918-930.

Evanno, G., Regnaut, S., & Goudet, J. (2005). Detecting the number of clusters of individuals using the software STRUCTURE: a simulation study. *Molecular Ecology*, **14**, 2611-2620.

Exposito-Alonso, M. (2017). rbioclim: improved getData function from the raster R package to interact with past, present and future climate data from worldclim. org. *Available from: github.com/MoisesExpositoAlonso/rbioclim*.

Fick, S. E., & Hijmans, R. J. (2017). WorldClim 2: new 1‐km spatial resolution climate surfaces for global land areas. *International Journal of Climatology*, **37**, 4302-4315.

Lehnert, S. J., Bentzen, P., Kess, T., Lien, S., Horne, J. B., Clement, M., & Bradbury, I. R. (2019). Chromosome polymorphisms track trans-Atlantic divergence and secondary contact in Atlantic salmon. *Molecular Ecology*, **28**, 2074-2087.

Lehnert, S. J., Bradbury, I. R., April, J., Wringe, B. F., Van Wyngaarden, M., & Bentzen, P. (2023). *Pre-COSEWIC Review of Anadromous Atlantic Salmon (Salmo salar) in Canada, Part 1: Designatable Units*. DFO Can. Sci. Advis. Sec., Res. Doc. 2023/026.

Luu, K., Bazin, E., & Blum, M. G. (2017). pcadapt: an R package to perform genome scans for selection based on principal component analysis. *Molecular Ecology Resources*, **17**, 67-77.
